# Histopathological spectrum of common aldosterone-driver gene mutations in aldosterone-producing adenomas

**DOI:** 10.3389/fmed.2025.1569619

**Published:** 2025-06-10

**Authors:** Fatin Athirah Pauzi, Muaatamarulain Mustangin, Geok Chin Tan, Ales Ryska, Jiri Ceral, Miroslav Solar, Elena Aisha Azizan

**Affiliations:** ^1^Faculty of Medicine, National University of Malaysia, Kuala Lumpur, Malaysia; ^2^Charles University and University Hospital Hradec Kralove, Hradec Kralove, Czechia

**Keywords:** aldosterone-producing adenomas, CYP11B2-guided sequencing, aldosterone-driver mutations, genotype–phenotype correlations, immunohistochemistry analysis

## Abstract

Past studies on common mutant aldosterone-producing adenomas (APAs) had found genotype–phenotype correlations associated with histological appearance. Most of these studies did not perform CYP11B2-guided sequencing of APAs or sequencing for all the currently known aldosterone-driver genes. Hence, misinterpretation of the genotype–phenotype correlations could have occurred. Herein, we aimed to identify the genotype–phenotype correlations associated with the histopathology of the different mutant APAs utilizing CYP11B2-guided sequencing. A total of 33 APAs with confirmed aldosterone-driver mutation (17 *KCNJ5* mutant APAs, 8 *ATP1A1* mutant APAs, 6 *CACNA1D* mutant APAs, and 2 *CTNNB1* mutant APAs) were immunohistochemically stained using H&E, CYP17A1, CYP11B2, KCNJ5, Ki67, β-catenin, and LHCGR antibody. Interestingly, APAs with a p.Thr41Ala *CTNNB1* mutation also harbored a p.Val1373Met *CACNA1D* mutation. The *CTNNB1* double mutant APAs had less expression of CYP17A1 and larger quantities of spironolactone bodies than a single mutant APA with a p.Ser45Phe *CTNNB1* mutation. However, both *CTNNB1* mutant APAs displayed diffuse active β-catenin expression with prominent nuclear staining that reflects the constitutive activation of the Wnt/β-catenin signaling pathway (*p* = 0.016 compared to other genotypes) but no significant increase in LHCGR. *KCNJ5* mutant APAs displayed distinct existence of atypical cells (6 of the 17 *KCNJ5* mutant APAs), whereas *CACNA1D* mutant APAs had frequent presentations of spironolactone bodies (4 of the 6 *CACNA1D* mutant APAs), and *ATP1A1* mutant APAs had significantly higher Ki67 score than *KCNJ5* mutant APAs (*p* = 0.020). The results of this study support the notion that CYP11B2-guided sequencing of all currently known aldosterone-driver genes can fine-tune existing genotype–phenotype correlations in histopathological profiles.

## 1 Introduction

Primary aldosteronism (PA), also known as Conn’s syndrome, is one of the most common and potentially curable forms of secondary hypertension, also referred to as endocrine hypertension (1). PA can be caused by the autonomous aldosterone production in one or both adrenal glands, leading to elevated aldosterone levels despite suppressed renin activity (2). In nearly half of PA cases, the identification of a unilateral aldosterone-producing adenoma (APA) or aldosterone-producing nodule (APN) offers the opportunity for a complete cure of hypertension through the removal of the autonomous aldosterone-producing lesion (3). Studies on excised tissue have identified gain-of-function somatic mutations as the cause of autonomous aldosterone production (4–8). The most frequent mutations were found in the genes *KCNJ5*, *CACNA1D*, and *ATP1A1* (8–11). In addition to the more common aldosterone-driver mutations, past studies have also identified recurring aldosterone-driver somatic mutations in the genes *ATP2B3*, *CTNNB1* (with or without *GNA11/GNAQ*), *CACNA1H*, *CLCN2*, *CADM1,* and *SLC30A1* in APAs (12–18).

The majority of the aldosterone-driver genes encode for ion channels or pumps (*KCNJ5*, *CACNA1D*, *CACNA1H*, *ATP1A1*, *ATP2B3,* and *SLC30A1*) that are involved in regulating intracellular ion homeostasis and plasma membrane potential (9). In general, mutations of these genes lead to the depolarization of the cell membrane due to the impairment of ion transport that activates voltage-gated Ca^2+^ channels. This alteration consequently increases intracellular calcium levels and promotes the transcription of aldosterone synthase (CYP11B2), leading to excessive aldosterone biosynthesis. Thus, adrenal immunohistochemistry (IHC) for CYP11B2 could detect cells that can synthesize aldosterone, enabling non-functional nodules and adenomas co-existing with those that synthesize aldosterone to be distinguished. This has only been made possible in the last decade, with the successful development of a specific monoclonal antibody for CYP11B2 that can differentiate from the highly homologous CYP11B1 (19). To note, CYP11B2-guided sequencing has a higher detection rate of aldosterone-driver mutations than non-CYP11B2-guided sequencing (20).

Past studies on the excised tissue of mutant APAs have found genotype–phenotype correlations associated with the morphologic appearance (4–6, 8, 21). However, many of these studies did not perform CYP11B2-guided sequencing or sequencing for all the currently known aldosterone-driver genes (most performed was *KCNJ5* genotyping). Thus, one could postulate that past studies that had not performed CYP11B2-guided sequencing or performed genotyping of all aldosterone-driver genes could have misinterpreted the genotype–phenotype correlations observed.

Herein, this study aimed to identify the genotype–phenotype correlations associated with the histology of APAs with different mutations utilizing CYP11B2-guided sequencing. Correlation with histological features was performed using IHC staining of CYP17A1 and KCNJ5. CYP17A1 is usually not present in the ZG as its main physiological expression is in the zona fasciculata (ZF) and zona reticularis (ZR) in the adrenal cortex where it converts progesterone to 17α-(OH) progesterone to produce glucocorticoids, or pregnenolone to 17α-(OH) pregnenolone to produce sex steroid precursors (22). KCNJ5 is expressed more in the ZG, though its exact role in the adrenal gland is yet to be defined (23). The proliferation marker Ki67 was used to estimate the proliferation rate of the mutant adenoma. Furthermore, β-catenin nuclear expression was utilized as a measure of constitutive activation of Wnt/β-catenin signaling, and LHCGR expression was measured as *CTNNB1.* Mutant APAs that harbored a GNA11 or GNAQ aldosterone-driver mutation have been reported to have increased LHCGR expression and onset of disease during high periods of HCG (4, 17, 24).

## 2 Methods

### 2.1 Sample acquisition

Adrenal tissues were collected from 66 Czech patients with unilateral PA who underwent adrenalectomy at the University Hospital Hradec Kralove. All the patients gave written informed consent. The study was approved by the University Hospital Hradec Kralove Ethics Committee (201504 S22P) and the National University of Malaysia Research Ethics Committee (FF-2015-092). Case detection and subtype identification were in accordance with local guidelines described in detail previously (25, 26). Confirmation and lateralization of PA were performed as detailed in Supplementary material.

### 2.2 Genotyping of somatic mutations in APAs

APA lesion was identified in formalin-fixed paraffin-embedded (FFPE) adrenal sections of unilateral PA patients through positive immunohistochemistry (IHC) staining of CYP11B2. CYP11B2 IHC-guided DNA extraction of adenoma tissue was then performed on 3–5 serial sections of 10 μm thickness using a commercial DNA extraction kit to ensure the extraction of APA tissue that expressed aldosterone synthase. Genotype of the APA DNA was determined through targeted sequencing of aldosterone-driver mutation hotspots as previously reported (8, 27) or through commercial targeted sequencing of aldosterone-driver genes (*KCNJ5*, *ATP1A1*, *ATP2B3*, *CACNA1D*, and *CTNNB1*) using the DNBSEQ platform powered by combinatorial Probe-Anchor Synthesis (cPAS) and improved DNA Nanoballs (DNB) technology (BGI Genomics Co., Ltd., Hong Kong). The cPAS chemistry works by linking a fluorescent probe to a DNA anchor on the DNB, followed by high-resolution digital imaging. Sequencing-derived raw image files were processed by a DNBSEQ base-calling software for base-calling with default parameters, and the sequence data of each individual was generated as paired-end reads, which were defined as “raw data” and stored in FASTQ format.

A total of 33 APAs had a known aldosterone-driver mutation in the aldosterone-driver genes *KCNJ5*, *ATP1A1*, *CACNA1D*, and *CTNNB1*, with sufficient material for further immunohistochemical analysis. A total of 24 had been characterized previously (8, 27), of which 3 samples initially classified as wild-type by targeted sequencing of aldosterone-driver mutation hotspots were, in this study, re-classified as having a known aldosterone-driver mutation based on targeted sequencing of aldosterone-driver genes using the DNBSEQ platform. Mutations identified through the DNBSEQ platforms were then annotated and filtered using Polymorphism Phenotyping v2 (PolyPhen-2 v2.2.2 build r394) (28) and Sorting Intolerant From Tolerant (SIFT v1.03) (29). The combined prediction results were considered evidence supporting a deleterious effect of a variant. Variants were also characterized using the Mutation Assessor (30), the Mutation Taster (31), and the Combined Annotation Dependent Depletion (CADD) score (32).

### 2.3 Immunohistochemistry (IHC) staining

IHC staining was performed on FFPE tissue sections using the detection system EnVision FLEX+, Mouse, High pH (Dako, Denmark) according to the manufacturer’s recommendations. IHC staining for CYP11B2 was performed on all samples, and then sections that had a positive nodule with CYP11B2 were stained with other antibodies (Supplementary Table S1). IHC staining of CYP11B2 and β-catenin was performed using selective mouse monoclonal antibodies, whereas staining of CYP17A1, KCNJ5, and LHCGR was performed using rabbit polyclonal antibodies. The antibodies for CYP11B2 and CYP17A1 were provided as a gift by Prof Celso Gomez-Sanchez (University of Mississippi Medical Center, USA). Staining of Ki67 was performed using a commercial ready-to-use mouse monoclonal antibody (Clone MIB-1, Catalog No. IR62661, Dako, Denmark). Details of IHC staining protocols are described in Supplementary Table S1. Positive control tissues were stained with every batch, ensuring staining was specific and selective in the IHC experiments (Supplementary Figure S1).

Scoring of IHC staining for CYP11B2, CYP17A1, KCNJ5, and LHCGR was performed genotype-blinded, with a score of 0 representing 0% expression to 10 representing 100% expression (further details of the score are provided in Supplementary Table S2; Supplementary Figures S2–S6). As for β-catenin immunostaining, the level of nuclear staining was determined by scoring as detailed in Supplementary Table S3. Intense Ki67 nuclei staining of representative fields of the APAs’ histology was photographed and quantified using Image J with a previously published semi-automated image analysis of high-contrast tissue areas method (detailed in Supplementary material; Supplementary Figure S7) (27, 33). The percentage of atypical cells and spironolactone bodies was determined from three representative images of the APAs’ histology using hematoxylin and eosin-stained (H&E) sections.

### 2.4 Statistical analysis

All data are presented as mean±SEM for the indicated number of experiments (*n*) unless specified otherwise as median (IQR). Statistical analysis was performed using standard statistical software, and the statistical significance was set at a *p*-value of <0.05. Assessment of the normality of data was analyzed using the Shapiro–Wilk test, in which comparisons of normally distributed data were performed using the independent *t*-test. Non-normally distributed data and ordinal data were compared using the Mann–Whitney U-test. Comparison of categorical data was analyzed using Fisher’s exact test.

## 3 Results

### 3.1 CYP11B2-guided sequencing of APAs

**Figure 1:**
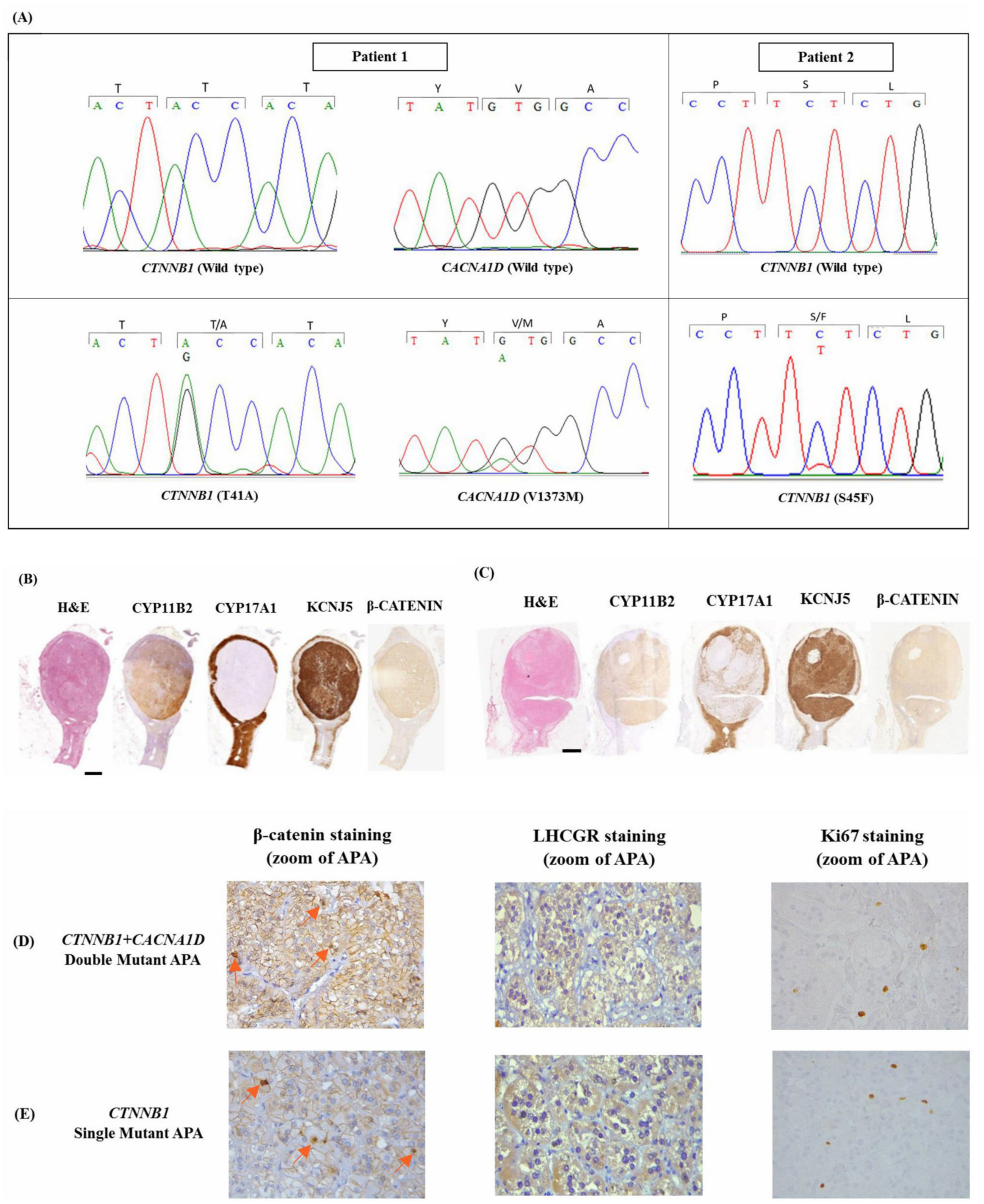
**(A)** DNA of patient 1 with mutations *CTNNB1* T41A and *CACNA1D* V1373M, and patient 2 with mutation *CTNNB1* S45F. Mutations were found in the adenoma but not the adjacent adrenal gland. APA, aldosterone-producing adenoma. **(B)** Immunohistochemical characterization of APAs harboring a *CTNNB1* and *CACNA1D* pathogenic mutation or **(C)** just a *CTNNB1* pathogenic mutation. Scale bars, 2 mm. **(D)** Comparison of β-catenin, LHCGR and Ki67 IHC staining of *CTNNB1* and *CACNA1D* double mutant APA and **(E)** *CTNNB1* single mutant APA. Orange arrows indicate nuclear staining.* CTNNB1 and CACNA1D.

Of the 33 APA samples with sufficient material for genotype–phenotype analysis, 17 APAs had an aldosterone-driver mutation in *KCNJ5*, 8 APAs in *ATP1A1*, 6 APAs in *CACNA1D,* and 2 APAs in *CTNNB1*. Of particular interest, one *CTNNB1* mutant APAs harbored two known aldosterone-driver mutations, the p.Thr41Ala *CTNNB1* mutation and the p.Val1373Met *CACNA1D,* which was confirmed by Sanger Sequencing (Figure 1A). The variants replaced an A with a G (c.121A > G) in *CTNNB1*, resulting in the p.Thr41Ala substitution, and replaced a G with an A (c.4117G > A) in *CACNA1D*, resulting in the p.Val1373Met substitution. These variants were somatic, only present in the CYP11B2-positive APA tissue but not in the adjacent normal adrenal (Figure 1A). The SIFT score of both mutations was close to 0 (a SIFT score of 0–0.05 predicts an intolerable mutation that can affect protein function), whereas PolyPhen-2 was close to 1 (which also predicts a deleterious variant) (Supplementary Table S4). Similarly, Mutation Assessor with an FI score greater than 2.00 (*CTNNB1*; 2.68 (M) and *CACNA1D*; 3.62 (H)) and Mutation Taster scores closer to 1 (*CTNNB1*; 1 (D) and *CACNA1D*; 1 (D)) predicted both these mutations as ‘deleterious’ (Supplementary Table S5). These values support the possibility that these *CACNA1D* and *CTNNB1* mutations are both likely pathogenic for APA pathology.

Clinical characteristics of patients’ pre-adrenalectomy for each genotype are compared in Table 1. Although there was no significant difference in gender by genotype, the majority of female PA patients (7 of 9) harbored a *KCNJ5* mutant APAs. This high prevalence mutation also significantly occurred in younger patients, in contrast to the other somatic mutations that were frequently detected in older male patients (*p* = 0.002; Table 1). Moreover, patients with *KCNJ5* mutant APAs (n = 17) were on a significantly lower number of antihypertensive medications compared to other mutants (*n* = 16; 3 ± 0.4 vs. 5 ± 0.3; *p* = 0.016), indicating less resistant hypertension, and having a more pronounced suppression of renin (plasma active renin 2.9 ± 0.1 vs. 3.5 ± 0.3, *p* = 0.013). Other clinical characteristics listed in Table 1 were not significantly different between patients with or without a *KCNJ5* aldosterone-driver mutation. To note, in this cohort, the significant age difference was mainly driven by *ATP1A1* mutation, whereas the difference in plasma active renin was mainly driven by *CACNA1D* mutation (Table 1).

**Table 1:** Clinical characteristics of patients pre-adrenalectomy.

Characteristics	*KCNJ5* (n = 17)	*ATP1A1* (n = 8)	*CACNA1D* (n = 6)	*CTNNB1* double mutants (n = 1)	*CTNNB1* single mutant (n = 1)	*p-*value *KCNJ5* vs. others	*p-*value *KCNJ5* vs. *ATP1A1*	*p-*value *KCNJ5* vs. *CACNA1D*	*p-*value *ATP1A1* vs. *CACNA1D*
Sex (male:female)	10:7	7:1	6:0	1:0	0:1	0.118	0.205	0.124	1.000
Age	43 ± 2	59 ± 3	48 ± 5	53	62	0.002	0.000	0.317	0.038
SBP, mmHg	142 ± 5	147 ± 11	141 ± 9	130	128	0.934	0.603	0.933	0.687
DBP, mmHg	88 ± 3	86 ± 6	91 ± 7	86	88	0.946	0.669	0.657	0.575
Number of AH meds	3 ± 0.4	4 ± 0.5	5 ± 0.6	5	5	0.016	0.133	0.056	0.586
Serum Na, mmol/L	140 ± 0.6	140 ± 0.8	141 ± 1.6	143	142	0.667	0.936	0.877	0.942
Serum K, mmol/L	3.7 ± 0.1	3.8 ± 0.2	4.0 ± 0.3	3.7	3.9	0.345	0.655	0.300	0.524
Serum aldosterone, pmol/L^a^	1,153 ± 132	1828 ± 739	886 ± 177	520	470	0.358	0.884	0.293	0.302
Plasma active renin, ng/L^b^	2.9 ± 0.1	3.5 ± 0.5	3.7 ± 0.4	3.6	3.2	0.013	0.168	0.011	0.335

Data are shown as mean±SEM. Statistics on continuous variables were performed with the independent *t*-test for normally distributed data, and the Mann–Whitney U-test for non-normally distributed data. A comparison of sex between genotypes was performed using Fisher’s exact test. DBP, diastolic blood pressure; SBP, systolic blood pressure; AH meds, antihypertensive medications; Na, sodium; K, potassium; NS, not significant (*p*-value > 0.05). Statistical comparison of the clinical characteristics for *CACNA1D* did not include patients who had *CTNNB1* and *CACNA1D* mutants.

a Serum aldosterone values in ng/dL were converted to pmol/L using a conversion factor of 27.74 (44).

b 1 plasma active renin data from a patient with KCNJ5 mutation was excluded from the analysis as the value is affected by spironolactone due to the patient having severe hypertension complicated by aortic dissection, and treatment withdrawal is not possible.

### 3.2 Clinical presentation of the double mutant APA

The *CACNA1D/CTNNB1* double mutant APAs were adrenalectomized from a 53-year-old male patient. He had been referred for resistant arterial hypertension and a history of hypokalemia (serum potassium of 3.7 mmol/L, Table 1). A screening test for PA was performed when the patient was on verapamil and doxazosin with potassium supplementation. The aldosterone–renin ratio measured then was 80. The saline infusion confirmatory test was positive, with serum aldosterone measuring 200 pmoL/L at the end of infusion after sodium loading. AVS was then performed, and unilateral PA was diagnosed. A computed tomography scan was interpreted as adrenal hyperplasia, although histopathological analysis of the excised adrenal identified a 10-mm nodule (Figure 1B). Post-adrenalectomy of the affected adrenal gland, the blood pressure was controlled (124/84 mmHg) on doxazosin 4 mg, amlodipine 5 mg, and telmisartan 80 mg (Supplementary Table S6).

For comparison, APAs detected to have a single mutation of *CTNNB1* (p.S45F) were used. The variant replaced a C with a T (c.134C > T) as shown in Figure 1A. Mutant APAs were adrenalectomized from a 62-year-old woman. She also had resistant arterial hypertension and a history of hypokalemia. A screening test performed while the patient was on verapamil and doxazosin, with potassium supplementation, measured an aldosterone–renin ratio of 223. A saline infusion confirmatory test measured serum aldosterone of 1,260 pmoL/L at the end of infusion (after sodium loading). AVS was performed, and unilateral PA was diagnosed. Like double mutant APAs, the histopathological analysis identified an 11-mm nodule, although the computed tomography scan reported adrenal hyperplasia (Figure 1C). She underwent laparoscopic adrenalectomy of the affected adrenal, which improved her blood pressure (110/66 mmHg), though she still requires some antihypertensive medications (amlodipine 5 mg and telmisartan 80 mg) (Supplementary Table S6).

### 3.3 Histopathologic features of APAs somatic mutation

The histopathologic features of each variant were identified using H&E staining and immunohistochemical detection of CYP17A1, CYP11B2, KCNJ5, Ki67, β-catenin, and LHCGR (Supplementary Figures S8, S9). All the mutant APAs exhibited moderate to high expression of CYP11B2, with significantly higher IHC scores in *ATP1A1* and *CACNA1D* mutant APAs than *KCNJ5* mutant APAs (7 [6–9] and 8 [6–9] vs. 6 [5–6]; *p* < 0.05; Table 2). In contrast to the *KCNJ5* mutant APAs, *ATP1A1*, *CACNA1D*, and *CTNNB1* mutant APAs had a significantly lower score of CYP17A1 expression (2 [1–4] vs. 7 [6–7]; *p* ≤ 0.001), with higher score of KCNJ5 (9 [8–10] and 9 [8–9] vs. 4 [4–6]; *p* < 0.001; Table 2). Furthermore, the non-*KCNJ5* mutant APAs had a significantly higher Ki67 score than *KCNJ5* mutant APAs, which was mainly driven by *ATP1A1* mutation (0.061 ± 0.007 vs. 0.042 ± 0.005; *p* = 0.019; Table 2). Yet, the average adenoma size, based on the diameter, was significantly larger in *KCNJ5* mutant APAs compared to non-*KCNJ5*-mutant adenomas (13 ± 0.8 vs. 9 ± 0.9; *p* = 0.001; Table 2). To note, *CACNA1D* mutant APAs had a significantly lower β-catenin IHC score than *KCNJ5* mutant APAs (2 [1–2] vs. 2 [2–3]; *p* = 0.026; Table 2). However, this lower score in the *CACNA1D* mutant APAs represents less nuclear staining rather than lower cytoplasmic expression of β-catenin compared to *KCNJ5* mutant APAs. The positive nuclear β-catenin staining indicates that the Wnt/β-catenin signaling was active in these APAs. Whereas for the LHCGR, no significant difference was seen between groups (LHCGR score of *KCNJ5* mutant APAs: 2 (1–4), n = 7; *ATP1A1* mutant APAs: 3 (2–4), n = 3; *CACNA1D* mutant APAs: 3 (2–3), n = 3; *CTNNB1* double mutant APAs: 4, n = 1; *CTNNB1* single mutant APAs: 4, n = 1); however, the *CTNNB1* double and single mutant APAs did have the highest score.

**Table 2:** Comparison of cellular histochemistry by the APA genotype.

Characteristics	*KCNJ5* (n = 17)	*ATP1A1* (n = 8)	*CACNA1D* (n = 6)	*CTNNB1* double mutants (n = 1)	*CTNNB1* single mutant (n = 1)	*p-*value *KCNJ5* vs. others	*p-*value *KCNJ5* vs.*ATP1A1*	*p-*value *KCNJ5* vs.*CACNA1D*	*p-*value *ATP1A1* vs.*CACNA1D*
CYP11B2 score	6 (5–6)	7 (6–9)	8 (6–9)	4	4	0.005	0.001	0.023	0.895
CYP17A1score	7 (6–7)	2 (1–4)	2 (1–4)	1	3	0.000	0.000	0.001	0.838
KCNJ5 score	4 (4–6)	9 (8–10)	9 (8–9)	7	8	0.000	0.000	0.000	0.946
Ki67 score^a^	0.042 ± 0.005	0.063 ± 0.008	0.051 ± 0.014	0.080	0.084	0.019	0.020	0.438	0.400
β-catenin score^a^	2 (2–3)	2 (1–3)	2 (1–2)	4	4	0.220	0.163	0.026	0.266
Size of the adenoma, mm	13 ± 0.8	9 ± 1.5	8 ± 1.2	10	11	0.001	0.013	0.002	0.521
Atypical cells (Absent:Present)	6:11	8:0	6:0	1:0	1:0	0.000	0.003	0.014	ND
Spironolactone bodies (Absent:Present)	17:0	8:0	2:4	0:1	0:1	0.007	ND	0.002	0.015

Normal variables are shown as mean±SEM, and non-normal variables are shown as median (25%–75%). Statistics were performed with the independent sample *t*-test, Mann–Whitney U-test, and Fisher’s exact test. ND, Not detected.

a *KCNJ5* had two missing data for Ki67 score, *ATP1A1* had one missing data for Ki67 score, *CACNA1D* had one missing data for Ki67 score, and two missing data for β-catenin score.

Comparing the *CTNNB1* mutant APAs, both the double mutant and single mutant APAs exhibited comparable features in H&E staining, as well as similar expression of CYP11B2, KCNJ5, Ki67, β-catenin, and LHCGR (Table 2, Figures 1B–E, and Supplementary Figure S10). In both APAs harboring *CTNNB1* mutations, the β-catenin was diffusely expressed, demonstrating both cytoplasmic and strong nuclear expression, compared to the other mutant APAs that displayed weak nuclear and cytoplasmic β-catenin expression (4 vs. 2 [2–3]; *p* = 0.016; Table 2 and Supplementary Figure S10). To note, *CTNNB1* and *CACNA1D* double mutant APAs did have a lower CYP17A1 score than single *CTNNB1* mutant APAs (1 vs. 3, Table 2 and Figures 1D,E).

**Figure 2:**
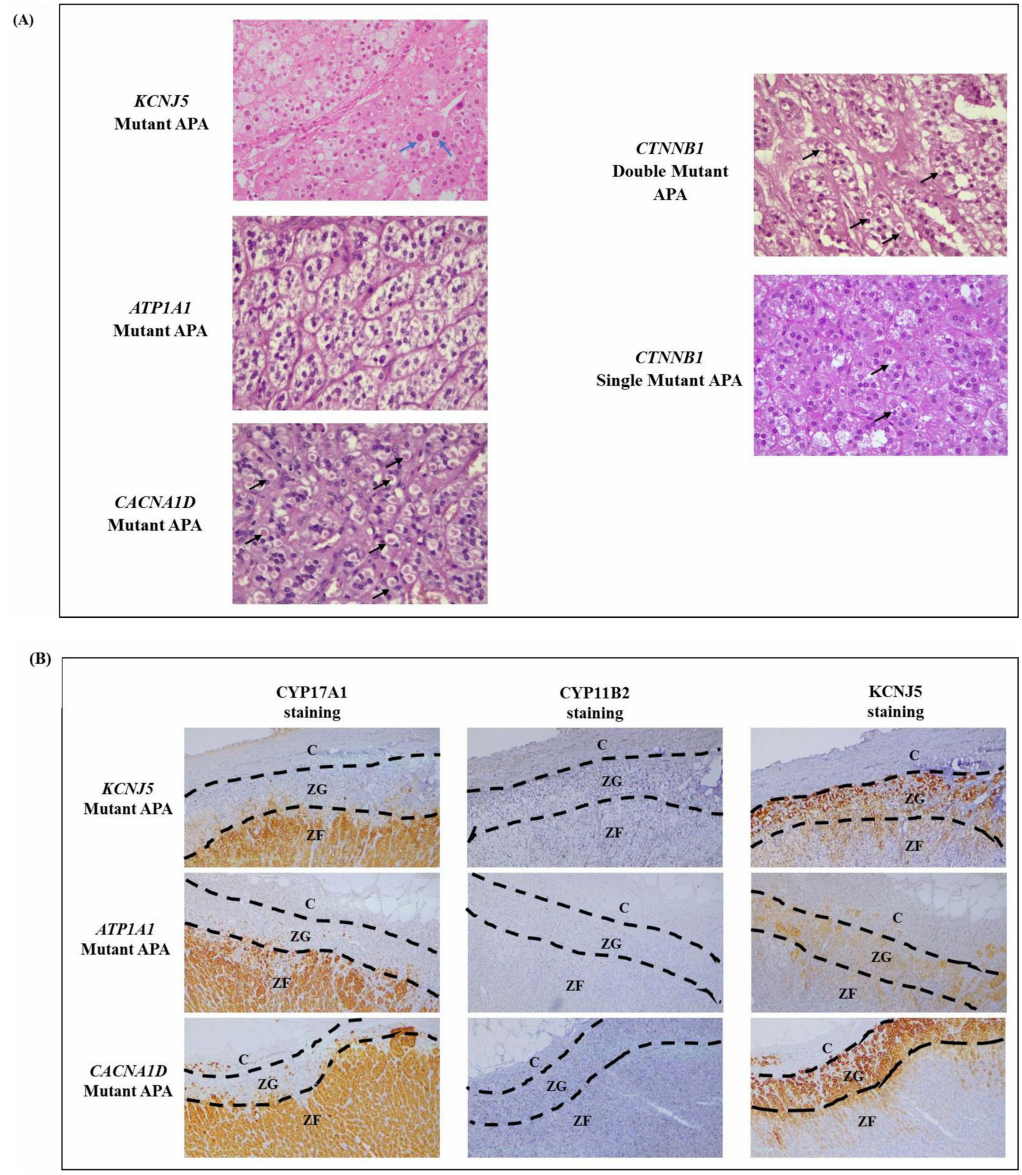
**(A)** Representative H&E staining of mutant APAs. Black arrows point to spironolactone bodies, and blue arrows point to atypical nuclei. **(B)** Zona glomerulosa hyperplasia as determined by a lack of CYP17A1 IHC staining and intense KCNJ5 IHC staining.

Another significant observation from the immunohistochemistry analysis is the histopathologic findings of spironolactone bodies and atypical cells (Table 2; Supplementary Table S7). The spironolactone bodies, detected by H&E staining as round, laminated cytoplasmic inclusions surrounded by a clear halo, were often found in cells with a low cytoplasmic to nucleus ratio, similar to those of the zona glomerulosa of the normal adrenal cortex (Figure 2A). These spironolactone bodies were observed frequently in *CACNA1D* and *CTNNB1* mutant APAs (Table 2; Supplementary Table S7). Interestingly, compared to *CTNNB1* single mutant APAs, spironolactone bodies were found in larger quantities in APAs with both *CTNNB1* and *CACNA1D* double mutations (Figure 2A). Conversely, atypical cells with large nuclei and prominent nucleoli were found only in *KCNJ5* mutant APAs, while other mutant APAs had monomorphic bland nuclei (Table 2 and Figure 2A). To note, ZG with positive CYP11B2 staining was detectable in the adrenals adjacent to *ATP1A1* mutant APAs (*n* = 2) and *KCNJ5* mutant APAs (*n* = 4; Supplementary Figure S11). The ZG region was determined based on the lack of CYP17A1 expression and intense KCNJ5 pericapsular immunohistochemical positivity. Interestingly, ZG hyperplasia had a trend to be thicker in *KCNJ5* mutant APAs (n = 8; 0.32 ± 0.03 mm) compared to other genotypes (n = 15; 0.28 ± 0.02 mm; *p* = 0.137, Figure 2B).

## 4 Discussion

Herein, two APAs with pathogenic *CTNNB1* mutations were found, with one also harboring a pathogenic *CACNA1D* mutation (p.V1373M). Somatic mutations in *CTNNB1* have been scarcely identified in PA patients, suggesting a low prevalence (4, 15). The majority of APAs with *CTNNB1* pathogenic mutations occur mutually exclusive to other aldosterone-driver somatic mutations, though there were rare reports of *CTNNB1* mutations existing with the co-driver mutations in *GNA11* and *GNAQ* that activated aldosterone production (17, 20, 34). This is different from the finding of micronodules harboring a pathogenic aldosterone-driving mutation in a separate gene from that of APAs in the same adrenal (20, 34). The rarity of APAs with double pathogenic mutations (in this case, *CTNNB1* and *CACNA1D*) makes their immunohistochemical and biochemical phenotype of interest. The identified variants in *CTNNB1*, p.T41A, and *CACNA1D*, p.V1373M, have both been previously found separately in APAs (4, 7, 35). Supporting further its pathogenic nature, several algorithm tools (SIFT, PolyPhen-2, Mutation Assessor, and MutationTaster) identified both missense variants as damaging with functional consequences. Moreover, the sequencing result of the patient’s germline DNA confirmed these variants to be somatic. Therefore, the coexistence of these variants should be considered the presence of two independent concurrent driver mutations harbored by APAs.

This study characterized the histopathologic spectrum of identified somatic mutations in APAs using H&E staining and CYP17A1, CYP11B2, KCNJ5, Ki67, β-catenin, and LHCGR IHC. When comparing the *CTNNB1* mutant APAs, both single and double mutant APAs had a seemingly similar histopathologic phenotype, except that *CTNNB1* single mutant APAs had a higher expression of CYP17A1 compared to *CTNNB1* double mutant APAs. This is in line with previous reports, which showed that APAs harboring the *CTNNB1* mutation could display heterogeneous CYP11B2 and CYP11B1 expression (15).

CTNNB1 expression is normally restricted to the ZG in the adrenal gland (36). Nuclear expression of CTNNB1 reflects the constitutive activation of the Wnt/β-catenin signaling pathway (37). During constitutive activation of Wnt/β-catenin signaling in the ZG, translocation of β-catenin to the nucleus is thought to increase expression of nuclear receptors NURR1 and NUR77, responsible for increased transcription of downstream targets including T-cell factor/lymphoid enhancer factor (TCF/LEF-1), CYP21, the Angiotensin I receptor (ATR1), and CYP11B2 (24, 38, 39). Activation of the associated Wnt/β-catenin pathway has also been reported to lead to a functional block in the ability of the ZG cells to transdifferentiate into ZF cells, leading to hyperplasia (40). In this study, APAs harboring *CTNNB1* mutations (both single and double) displayed diffuse active β-catenin expression higher than other mutant APAs with prominent nuclear staining. The finding of these *CTNNB1* mutant APAs along with the high expression of β-catenin, both in the nucleus and the cytoplasm, highlights the central role of the Wnt/β-catenin signaling pathway in the development of APAs. Thus, targeting this pathway may be an important approach in the treatment of unilateral PA.

Constitutive activation of the Wnt signaling pathway in ZG-like adenomatous cells has also been postulated to lead to dedifferentiation toward their common adrenal–gonadal precursor cell type and lead to aberrant expression of gonadal receptor LHCGR and/or GnRHR (4). However, both single- and double-mutant APAs did not exhibit any significant difference in the expression of LHCGR, unlike the previous study that reported LHCGR was upregulated more than 10-fold in double-mutant APAs harboring *CTNNB1* and *GNA11*/*GNAQ* mutations (17). Nevertheless, *ATP1A1* and *CACNA1D* mutant APAs did have low expression of β-catenin and LHCGR IHC staining, along with low CYP17A1 and high CYP11B2 and KCNJ5 expression, as previously documented (27).

Clinically, both patients with a *CTNNB1* and *CACNA1D* double mutant APAs and the single *CTNNB1* mutant APAs have not achieved complete post-adrenalectomy resolution of hypertension. This contrasts with the previous study that found 10 patients with double mutant APAs of *CTNNB1* and *GNA11/Q* to be completely cured after adrenalectomy (17). However, both patients harboring the *CTNNB1* mutations were >50 years old, and thus, age-related essential hypertension cannot be ruled out for the post-adrenalectomy residual hypertension seen. APAs carrying *CTNNB1* mutations have been previously reported to have a higher possibility of residual hypertension than other mutant APAs, albeit these were most likely single *CTNNB1* mutant APAs (41). Concurringly, in Wu et al.’s study, the eight *CTNNB1* mutant APAs found most frequently occurred in females of older age with relatively large adrenal lesions similar to our patient with a single mutant APA.

In summary, the results of this study support the suggestion that CYP11B2-guided sequencing of all the currently known aldosterone-driver genes fine-tunes current genotype–phenotype relationships identified in PA patients with APAs. In addition, this study identified a rare *CTNNB1* mutant APA with a likely pathogenic *CACNA1D* mutation. Although there were many overlaps in histopathologic features between the single and double *CTNNB1* mutant APAs, *CTNNB1* APAs with a *CACNA1D* mutation did have a distinct profile with lower expression of CYP17A1 and larger quantities of spironolactone bodies. Furthermore, *KCNJ5* mutant APAs were found once again to have a lower Ki67 proliferation index (27). This study found that the finding was mainly driven by the high expression of Ki67 in *ATP1A1* mutant APAs. Interestingly, although both patients with *CACNA1D* and *ATP1A1* mutations were on a high number of antihypertensive medications, which suggested that spironolactone was most likely part of their drug management, spironolactone bodies, which are believed to be the result of treatment with spironolactone (42) were only found in *CACNA1D* mutant APAs (and *CTNNB1* mutant APAs). Although the significance of the spironolactone bodies remains unclear, it is postulated that these structures represent a compensatory attempt on the part of the cell to produce more mineralocorticoid or storage of the steroids, as the spironolactone bodies are probably derived from the endoplasmic reticulum, which is considered capable of storing steroids (42, 43). Other clinical parameters of patients harboring an *ATP1A1* or *CACNA1D* mutation are quite closely matched (except for age); thus, the existence of spironolactone bodies only in the *CACNA1D* mutant APAs but not in *ATP1A1* mutant APAs warrants further investigation. Nevertheless, despite the study’s capability to fine-tune the current genotype–phenotype histopathology profiles by utilizing CYP11B2-guided sequencing, a larger sample size is required to support the significant observations associated with the genotypes. Moreover, functional characterization of the mechanism for the variants in double mutant APAs is also needed to confirm the pathogenicity of the variants.

## Data Availability

The contributions presented in the study are all publicly available. Original sequencing data can be found at PRJNA1269091 (SRA - NCBI) while previously reported sequencing data is published as cited in main article (DOI: 10.1161/HYPERTENSIONAHA.117.09057). The raw data supporting the conclusions of this article can be made available upon request, further inquiries can be directed to the corresponding author.
