# Supplementary material for: Histopathological spectrum of common aldosterone-driver gene mutations in aldosterone-producing adenomas
Source: Front Med (Lausanne). 2025 Jun 10;12:1569619. doi: 10.3389/fmed.2025.1569619 (PMC12185499; doi:10.3389/fmed.2025.1569619)
Supplement: Supplementary file 1 [file Data_Sheet_1.docx]

Supplementary Material

# Supplementary Methods

## Confirmation and lateralization of primary aldosteronism (PA)

The diagnosis of PA was confirmed by saline infusion testing. A definite adenoma on CT was not mandatory for diagnosis of unilateral PA. Instead, the lateralization of aldosterone secretion was diagnosed by adrenal venous sampling. Aldosterone secretion was considered lateralized, and adrenal surgery was indicated, when the cortisol corrected aldosterone concentration in the venous sample from the dominant adrenal gland was at least four times higher compared to the contralateral gland. In all enrolled patients adrenalectomy reversed the biochemical abnormalities and led to the improvement of blood pressure control.

## Quantification of intense Ki67 nuclei staining

3 images (x4 magnification, >500 cells) per aldosterone-producing adenoma (APA) were acquired to have digital morphometry performed on. These images were taken to best describe the tissue section as a whole. Morphometric procedures which include HueSaturation-Brightness (HSB)-based color segmentation were then performed using Image J as detailed elsewhere (33) . Particle analysis was then performed on areas that were “separated” (through color segmentation) from the original tissue based on their HSB characteristics (illustrated in Figure S6). The Ki67 score was calculated by taking the average of the 3 images Ki67 staining count divided by the hematoxylin staining (nucleus) count.

# Supplementary Figures and Tables

**Table S1.** List of primary antibodies and parameter used for IHC and H&E staining

| **No.** | **Primary Antibody** | **Source/ Product Code** | **Primary Antibody Dilution** | **Primary Antibody Incubation Time** | **Pre-treatment Conditions (Temperature / Time)** | **Positive Tissue Control** |
| --- | --- | --- | --- | --- | --- | --- |
| 1 | Rabbit polyclonal anti-KCNJ5 | Sigma-Aldrich Cat# HPA017353, RRID:AB_1852135 | 1:100 | 20 minutes | 95°C /  20 minutes | Normal adrenal |
| 2 | Rabbit polyclonal anti-17α-hydroxylase | *NA-gift from Prof Celso Gomez-Sanchez | 1:200 | 20 minutes | 95°C /  20 minutes | Normal adrenal  and testis |
| 3 | Mouse monoclonal anti-CYP11B2 | *NA-gift from Prof Celso Gomez-Sanchez | 1:100 | 60 minutes | 98°C /  30 minutes | Normal adrenal |
| 4 | Mouse monoclonal anti-Ki67 | Agilent Cat# IR626, RRID:AB_2890068 | Ready to use | 60 min | 98°C /  60 min | Tonsils |
| 5 | Mouse monoclonal anti-β-Catenin | BD Biosciences Cat# 610154, RRID:AB_397555 | 1:300 | 30 minutes | 110°C /  30 minutes | Breast carcinoma |
| 6 | Rabbit polyclonal anti-LHCGR | Novus Cat# NLS 1436, RRID:AB_525379 | 1:200 | 30 minutes | 110°C /  30 minutes | Breast carcinoma |

**Table S2.** Description of immunohistochemistry score (CYP11B2, CYP17A1, KCNJ5 and LHCGR)

| **Description** | **Score** |
| --- | --- |
| 0% expression | 0 |
| Predominantly negative, <20% cytoplasmic/membrane expression | 1 |
| Predominantly negative with regional (20-40%) cytoplasmic/membrane expression | 2 |
| Weak to moderate cytoplasmic expression in >50% of adenoma | 3 |
| Moderate cytoplasmic staining with small clusters of strong cytoplasmic staining | 4 |
| Strong membrane/cytoplasmic expression in 40-60% of adenoma | 5 |
| Variable weak to strong cytoplasmic staining in >70% of adenoma | 6 |
| Variable moderate to strong cytoplasmic expression in >70% of adenoma | 7 |
| Strong membrane/cytoplasmic in 70-80% of adenoma | 8 |
| Strong membrane/cytoplasmic in 90% of adenoma | 9 |
| Strong membrane/cytoplasmic in 100% of adenoma | 10 |

**Table S3.** Description of immunohistochemistry score (beta-catenin)

| **Description** | **Score** |
| --- | --- |
| No staining | 0 |
| Membranous/Cytoplasmic staining without nuclear staining | 1 |
| Membranous/Cytoplasmic staining with focal nuclear staining (<20% of nuclei) | 2 |
| Membranous/Cytoplasmic staining with diffuse faint nuclear staining (>20% of nuclei) | 3 |
| Membranous/Cytoplasmic staining with diffuse strong nuclear staining (>20% of nuclei) | 4 |

**Table S4.** In silico predictive algorithms score for variants found by TGS in the *CTNNB1* double mutant APA (sample 1) and *CTNNB1* single mutant APA (sample 2)

| **Samples** | **Gene** | **Transcript** | **Codon_Change** | **dbSNPv151_GRCh38** | **Ref** | **Obs** | **VAF** | **SIFT** | **PolyPhen-2** | **CADD** | **GERP** | **REVEL** |
| --- | --- | --- | --- | --- | --- | --- | --- | --- | --- | --- | --- | --- |
| Sample 1 | *CACNA1D* | ENST00000288139p.Val1373Met/ c.4117G>A | Gtg/Atg | NA | G | A | 0 | D  (0.0) | D  (1.0, 0.997, 0.999) | 7.048987 | 4.32 | 0.821 |
|  | *CTNNB1* | ENST00000349496p.Thr41Ala/ c.121A>G | Acc/Gcc | rs121913412 | A | G | 0 | D  (0.0,0.003, 0.002) | P  (0.694) | 5.437608 | 6.31 | 0.403 |
|  | *CADM1* | ENST00000331581p.Asp285Glu/ c.855T>G | gaT/gaG | rs45525440 | A | C | 0.1 | T  (0.504, 0.407, 0.496, 0.493, 0.482) | B  (0.014, 0.016, 0.056,0.021, 0.013) | 0.668641 | -3.56 | 0.142 |
|  | *CADM1* | ENST00000331581p.Val5Leu/ c.13G>C | Gtg/Ctg | rs112835318 | C | G | 0.1 | D (0.017,0.023, 0.016) | B  (0.019,0.087, 0.04,0.027) | 4.189859 | 3.73 | 0.095 |
|  | *KCNJ5* | ENST00000338350p.Gln282Glu/ c.844C>G | Cag/Gag | rs7102584 | C | G | 0.5 | T  (1.0) | B  (0.0) | 0.156779 | 2.35 | 0.323 |
|  | *CACNA1H* | ENST00000348261p.Arg2077His/ c.6230G>A | cGc/cAc | rs1054645 | G | A | 0.3 | T  (0.588) | B  (0.001, 0.0) | 0.919472 | -0.51 | 0.033 |
|  | *CACNA1H* | ENST00000348261p.Pro640Leu/ c.1919C>T | cCg/cTg | rs61734410 | C | T | 0.3 | T (0.778, 0.779) | B  (0.062, 0.019) | 1.381861 | 0.94 | 0.237 |
|  | *CACNA1H* | ENST00000348261p.Val664Ala/ c.1991T>C | gTc/gCc | rs4984636 | T | C | 0.2 | D  (0.011) | B, P (0.288, 0.501) | 3.495154 | 2.69 | 0.395 |
| Sample 2 | *CTNNB1* | ENST00000349496:p.Ser45Phe/c.134C>T | tCt/tTt | rs121913409 | C | T | 0 | D (0.0) | D (0.928) | 6.124876 | 6.31 | 0.353 |
|  | *CLCN2* | ENST00000265593:p.Arg73His/c.218G>A | cGc/cAc | rs144412275 | C | T | 0 | T (0.166,0.177,0.015,0.167) | B (0.003,0.002,0.001) | 2.080244 | 1.7 | 0.476 |
|  | *CADM1* | ENST00000331581:p.Asp285Glu/c.855T>G | gaT/gaG | rs45525440 | A | C | 0.1 | T (0.504,0.407,0.496,0.493,0.482) | B (0.014,0.016,0.056,0.021,0.013) | 0.668641 | -3.56 | 0.142 |
|  | *KCNJ5* | ENST00000338350:p.Gln282Glu/c.844C>G | Cag/Gag | rs7102584 | C | G | 0.5 | T (1.0) | B (0.0) | 0.156779 | 2.35 | 0.323 |
|  | *CACNA1H* | ENST00000348261:p.Arg2060His/c.6179G>A | cGc/cAc | rs1054644 | G | A | 0.2 | T (0.25) | B (0.001,0.004,0.005) | 1.046412 | -0.96 | 0.332 |
|  | *CACNA1H* | ENST00000348261:p.Glu310Lys/c.928G>A | Gag/Aag | rs758039020 | G | A | 0 | T (0.449,0.45) | P (0.828,0.827) | 4.860399 | 3.66 | 0.577 |
|  | *CACNA1H* | ENST00000348261:p.Met313Val/c.937A>G | Atg/Gtg | rs36117280 | A | G | 0.2 | T (0.672,0.673) | B (0.0) | -1.40275 | -5.39 | 0.274 |
|  | *CACNA1H* | ENST00000348261:p.Val664Ala/c.1991T>C | gTc/gCc | rs4984636  SIFT, Sorting Intolerant From Tolerant; PolyPhen2, Polymorphism Phenotyping v2; CADD, Combined Annotation Dependent Depletion; D, deleterious; P, possibly damaging; T, tolerated; B, benign. | T | C | 0.2 | D (0.011) | B,P (0.288,0.501) | 3.495154 | 2.69 | 0.395 |

**Table S5.** In silico predictive algorithms score for likely pathogenic variants found in APAs and APNs.

| **No** | **Gene** | **Exonic\|Biotype** | **Transcript** | **Impact** | **SIFT** | **PolyPhen-2** | **Mutation Assessor** | **MutationTaster** | **CADD** |
| --- | --- | --- | --- | --- | --- | --- | --- | --- | --- |
| 1 | *ATP1A1* | MISSENSE | ENST00000295598:p.Leu104Arg/c.311T>G | MODERATE | 0.0(D) | 0.999(D) | 5.01(H) | 1,1,1(D,D,D) | 6.752348 |
| 2 | *ATP1A1* | MISSENSE | ENST00000295598:p.Leu104Arg/c.311T>G | MODERATE | 0.0(D) | 0.999(D) | 5.01(H) | 1,1,1(D,D,D) | 6.752348 |
| 3 | *ATP1A1* | protein_coding | ENST00000295598:p.Glu960_Leu964delinsVal/c.2879_2890delAAGAGACAGCCC | MODERATE |  |  |  |  |  |
| 4 | *ATP1A1* | MISSENSE | ENST00000295598:p.Leu104Arg/c.311T>G | MODERATE | 0.0(D) | 0.999(D) | 5.01(H) | 1,1,1(D,D,D) | 6.752348 |
| 5 | *CACNA1D* | MISSENSE | ENST00000288139:p.Arg1010His/c.3029G>A | MODERATE | 0.0(D) | 1.0(D) | 4.895(H) | 0.99983,0.9997,0.99983(D,D,D) | 7.646239 |
| 6 | *CACNA1D* | MISSENSE | ENST00000288139:p.Phe767Val/c.2299T>G | MODERATE | 0.0(D) | 1.0,1.0,0.994,0.999(D) | 2.64(M) | 0.999998,0.999995,0.999998(D,D,D) | 6.165262 |
| 7 | *CACNA1D* | MISSENSE | ENST00000288139:p.Val1173Gly/c.3518T>G | MODERATE | 0.0(D) | 0.961,0.937,0.976,0.991,0.983(D) | 3.225(M) | 1,1,1,1(D,D,D,D) | 5.922513 |
| 8 | *CACNA1D* | MISSENSE | ENST00000288139:p.Phe767Leu/c.2301C>G | MODERATE | 0.001(D) | 0.999,0.99,0.998,0.998(D) | 2.33(M) | 0.999985,0.999965,0.999985(D,D,D) | 5.446025 |
| 9 | *CACNA1D* | MISSENSE | ENST00000288139:p.Phe767Leu/c.2301C>G | MODERATE | 0.001(D) | 0.999,0.99,0.998,0.998(D) | 2.33(M) | 0.999985,0.999965,0.999985(D,D,D) | 5.446025 |
| 10 | *CACNA1D* | MISSENSE | ENST00000288139:p.Val1373Met/c.4117G>A | MODERATE | 0.0(D) | 1.0,0.997,0.999,1.0,1.0(D) | 3.62(H) | 1,1,1,1(D,D,D,D) | 7.048987 |
| 11 | *CTNNB1* | MISSENSE | ENST00000349496:p.Thr41Ala/c.121A>G | MODERATE | 0.0,0.003,0.002,0.003,0.003,0.003,0.003,0.003,0.002(D) | 0.694(P) | 2.68(M) | 1,1,1,1,1(D,D,D,D,D) | 5.437608 |
| 12 | *CTNNB1* | MISSENSE | ENST00000349496:p.Ser45Phe/c.134C>T | MODERATE | 0.0(D) | 0.928(D) | 2.565(M) | 1,1,1,1,1(D,D,D,D,D) | 6.124876 |
| 13 | *KCNJ5* | MISSENSE | ENST00000338350:p.Leu168Arg/c.503T>G | MODERATE | 0.001(D) | 0.999(D) | 3.67(H) | 1,1,1(D,D,D) | 3.735074 |
| 14 | *KCNJ5* | MISSENSE | ENST00000338350:p.Leu168Arg/c.503T>G | MODERATE | 0.001(D) | 0.999(D) | 3.67(H) | 1,1,1(D,D,D) | 3.735074 |
| 15 | *KCNJ5* | MISSENSE | ENST00000338350:p.Leu168Arg/c.503T>G | MODERATE | 0.001(D) | 0.999(D) | 3.67(H) | 1,1,1(D,D,D) | 3.735074 |
| 16 | *KCNJ5* | MISSENSE | ENST00000338350:p.Gly151Arg/c.451G>C | MODERATE | 0.0(D) | 1.0(D) | 4.03(H) | 1,1,1(D,D,D) | 4.614395 |
| 17 | *KCNJ5* | MISSENSE | ENST00000338350:p.Gly151Arg/c.451G>C | MODERATE | 0.0(D) | 1.0(D) | 4.03(H) | 1,1,1(D,D,D) | 4.614395 |
| 18 | *KCNJ5* | MISSENSE | ENST00000338350:p.Gly151Arg/c.451G>A | MODERATE | 0.0(D) | 1.0(D) | 4.03(H) | 1,1,1(D,D,D) | 4.856137 |
| 19 | *KCNJ5* | MISSENSE | ENST00000338350:p.Gly151Arg/c.451G>A | MODERATE | 0.0(D) | 1.0(D) | 4.03(H) | 1,1,1(D,D,D) | 4.856137 |
| 20 | *KCNJ5* | MISSENSE | ENST00000338350:p.Gly151Arg/c.451G>A | MODERATE | 0.0(D) | 1.0(D) | 4.03(H) | 1,1,1(D,D,D) | 4.856137 |
| 21 | *KCNJ5* | MISSENSE | ENST00000338350:p.Leu168Arg/c.503T>G | MODERATE | 0.001(D) | 0.999(D) | 3.67(H) | 1,1,1(D,D,D) | 3.735074 |
| 22 | *KCNJ5* | MISSENSE | ENST00000338350:p.Gly151Arg/c.451G>A | MODERATE | 0.0(D) | 1.0(D) | 4.03(H) | 1,1,1(D,D,D) | 4.856137 |
| 23 | *KCNJ5* | MISSENSE | ENST00000338350:p.Leu168Arg/c.503T>G | MODERATE | 0.001(D) | 0.999(D) | 3.67(H) | 1,1,1(D,D,D) | 3.735074 |
| 24 | *KCNJ5* | MISSENSE | ENST00000338350:p.Leu168Arg/c.503T>G | MODERATE | 0.001(D) | 0.999(D) | 3.67(H) | 1,1,1(D,D,D) | 3.735074 |
| 25 | *KCNJ5* | MISSENSE | ENST00000338350:p.Leu168Arg/c.503T>G | MODERATE | 0.001(D) | 0.999(D) | 3.67(H) | 1,1,1(D,D,D) | 3.735074 |

SIFT, Sorting Intolerant From Tolerant; PolyPhen-2, Polymorphism Phenotyping v2; CADD, Combined Annotation Dependent Depletion; D, deleterious; P, possibly damaging; H, high deleterious probability, M, medium deleterious probability.

**Table S6.** Clinical characteristics of primary aldosteronism patients post-adrenalectomy

| **Patient ID** | **SBP (mmHg)** | **DBP (mmHg)** | **Number of AH meds** | **Serum Na (mmol/l)** | **Serum K (mmol/l)** | **Serum aldosterone (pmol/l)** | **Plasma active renin (ng/l)** |
| --- | --- | --- | --- | --- | --- | --- | --- |
| 1 | 126 | 64 | 5 | 144 | 4.1 | 280 | 106.9 |
| 2 | 130 | 80 | 3 | 143 | 5 | 550 | 44.8 |
| 3 | 126 | 82 | 1 | 140 | 5 | ND | ND |
| 4 | 136 | 90 | 0 | 140 | 4 | 290 | 25.9 |
| 5 | 110 | 76 | 3 | 141 | 4.2 | 490 | 69.3 |
| 6 | 116 | 80 | 1 | 141 | 4.3 | 140 | 5.9 |
| 7 | 120 | 76 | 4 | 138 | 5.2 | 100 | 142.4 |
| 8 | 126 | 80 | 4 | 137 | 4.3 | 70 | 16.1 |
| 9 | 122 | 76 | 2 | 139 | 4.5 | 70 | 11.7 |
| 10 | 126 | 82 | 4 | 141 | 4.7 | 255 | 42.6 |
| 11 | 132 | 90 | 2 | 132 | UN | 140 | 153 |
| 12 | 126 | 84 | 2 | UN | UN | 170 | 14.6 |
| 13 | 136 | 82 | 3 | 141 | 4.8 | 280 | 16.7 |
| 14 | 130 | 90 | 2 | 142 | 4.2 | UN | UN |
| 15 | 120 | 86 | 3 | 143 | 4 | 380 | 212 |
| 16 | 132 | 78 | 4 | 140 | 4.2 | 210 | 8.5 |
| 17 | 114 | 76 | 3 | 140 | 4.2 | 150 | 11.7 |
| 18 | 130 | 80 | 2 | 142 | 4.9 | 160 | 3.9 |
| 19 | 114 | 84 | 3 | 140 | 4.6 | 30 | 3 |
| 20 | 140 | 92 | 1 | 139 | 4.1 | 140 | 14.1 |
| 21 | 136 | 80 | 2 | 137 | 4.4 | 170 | 2.7 |
| 22 | 120 | 84 | 0 | 138 | 4 | 270 | 24.1 |
| 23 | 130 | 80 | 2 | 140 | 4.8 | 480 | 24.6 |
| 24 | 117 | 68 | 1 | 138 | 4.9 | 290 | 18 |
| 25 | 116 | 84 | 0 | 142 | 4.3 | 190 | 14.2 |
| 26 | 128 | 86 | 3 | 139 | 4.1 | 160 | 209.8 |
| 27 | 136 | 80 | 3 | 138 | 4.8 | 336 | 159.4 |
| 28 | 126 | 78 | 1 | 140 | 4 | 650 | 12 |
| 29 | 125 | 92 | 0 | 138 | 6 | ND | ND |
| 30 | 120 | 68 | 4 | 142 | 5 | 791 | 130 |
| 31 | 120 | 78 | 3 | ND | 4.5 | 170 | 12.7 |
| 32 | 130 | 86 | 3 | 138 | 4.3 | ND | ND |
| 33 | 128 | 90 | 2 | 140 | 4.6 | 200 | 30.3 |

DBP, diastolic blood pressure; SBP, systolic blood pressure; AH meds, antihypertensive medications; Na, sodium; K, potassium, UN, unknown; ND, not determined.

**Table S7.** Characterization of APA/APN cell histology from PA patients

| **Patient ID** | **Mutant Gene** | **Genotype** | **HISTALDO**  (3) | **Percentage of Atypical cells** | **Spironolactone bodies** |
| --- | --- | --- | --- | --- | --- |
| 1* | *ATP1A1* | L104R | APN | 0% | None |
| 2* | *ATP1A1* | L104R | APA | 0% | None |
| 3 | *ATP1A1* | L104R | APN | 0% | None |
| 4* | *ATP1A1* | F100_L104 del | APN | 0% | None |
| 5* | *ATP1A1* | L104R | APA | 0% | None |
| 6 | *ATP1A1* | F100_L104 del | APN | 0% | None |
| 7* | *ATP1A1* | F100_L104 del | APA | 0% | None |
| 8* | *ATP1A1* | L104R | APA | 0% | None |
| 9* | *CACNA1D* | R990H | APA | 0% | None |
| 10 | *CACNA1D* | F767V | APN | 0% | 16+10+5 |
| 11* | *CACNA1D* | V1153G | APN | 0% | None |
| 12* | *CACNA1D* | F747L | APN | 0% | 2+1+0 |
| 13 | *CACNA1D* | F767L | APA | 0% | 10+5+3 |
| 14* | *CACNA1D* | G403R | APN | 0% | Numerous >100 |
| 15 | *CACNA1D & CTNNB1* | V1373M/T41A | APA | 0% | Numerous >100 |
| 16 | *CTNNB1* | S45F | APA | 0% | 10+5+3 |
| 17* | *KCNJ5* | L168R | APA | <1% | None |
| 18* | *KCNJ5* | L168R | APA | <1% | None |
| 19 | *KCNJ5* | L168R | APA | 3-5% | None |
| 20* | *KCNJ5* | L168R | APA | 3-5% | None |
| 21* | *KCNJ5* | G151R | APA | 0% | None |
| 22* | *KCNJ5* | G151R | APA | 1-2% | None |
| 23* | *KCNJ5* | G151R | APA | <1% | None |
| 24* | *KCNJ5* | G151R | APA | 3-5% | None |
| 25* | *KCNJ5* | G151R | APA | 0% | None |
| 26* | *KCNJ5* | G151R | APA | 0% | None |
| 27 | *KCNJ5* | G151R | APA | 0% | None |
| 28 | *KCNJ5* | G151R | APN | <1% | None |
| 29 | *KCNJ5* | L168R | APA | 0% | None |
| 30 | *KCNJ5* | G151R | APA | <1% | None |
| 31 | *KCNJ5* | L168R | APA | 10% | None |
| 32 | *KCNJ5* | L168R | APA | 0% | None |
| 33* | *KCNJ5* | L168R & E145K | APA | 1-2% | None |

*The mutation status of the APAs within these patients have been reported before in Azizan et al. (2013) and Tan et al. (2017).


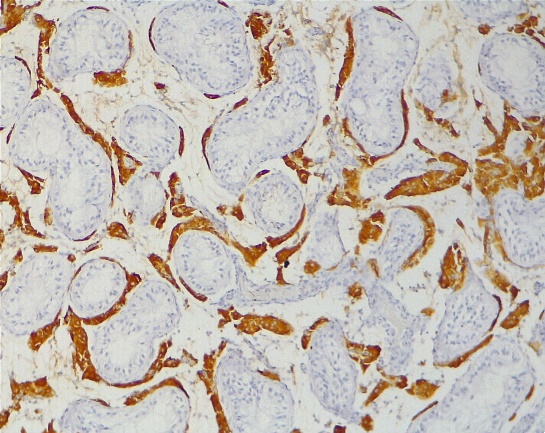


**(A)**

**(B)**

**(C)**

**(D)**

**(E)**

**(F)**


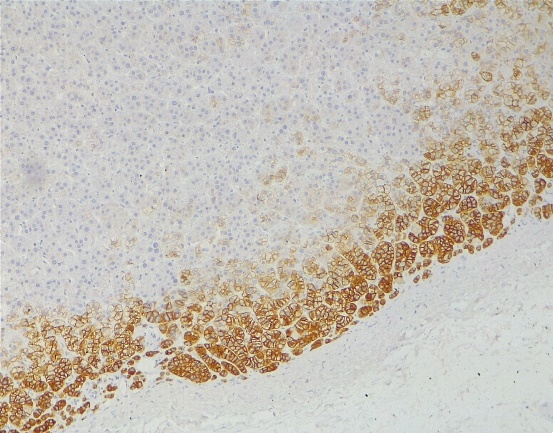

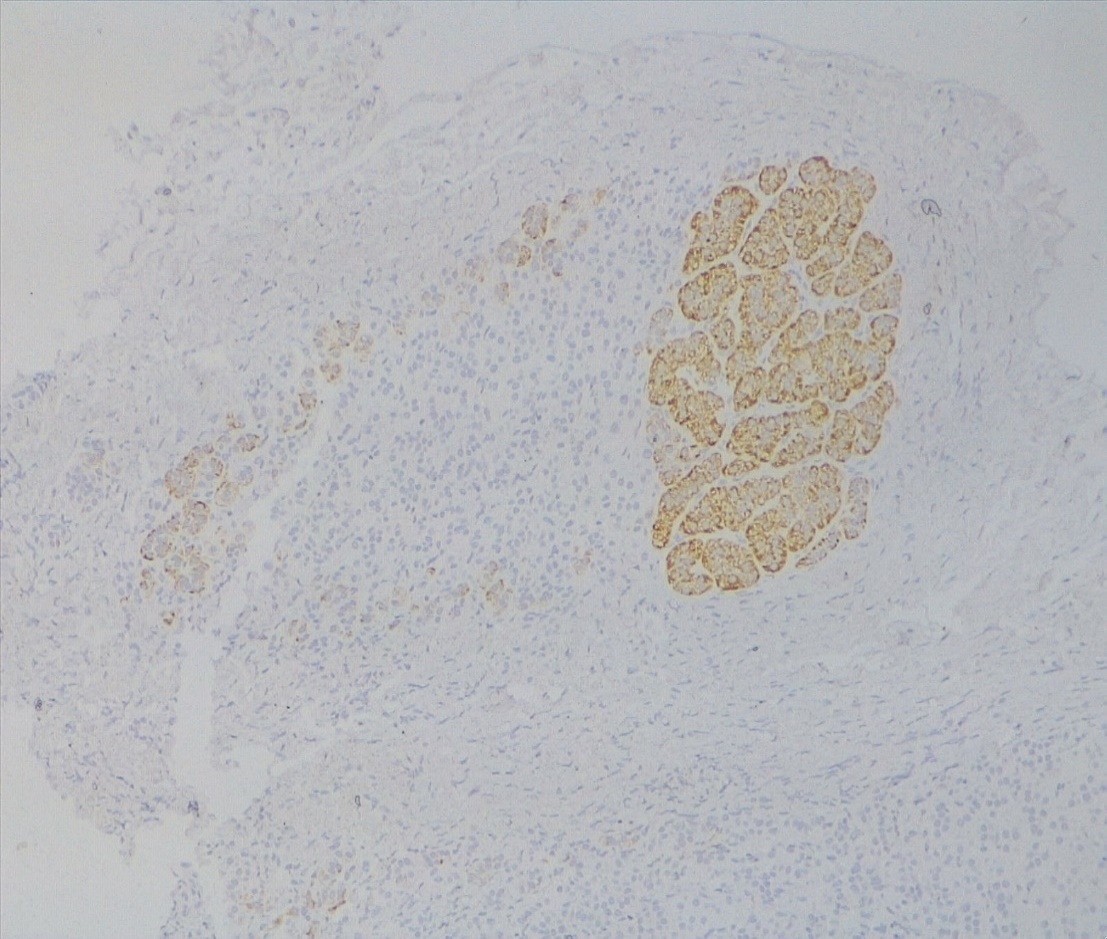


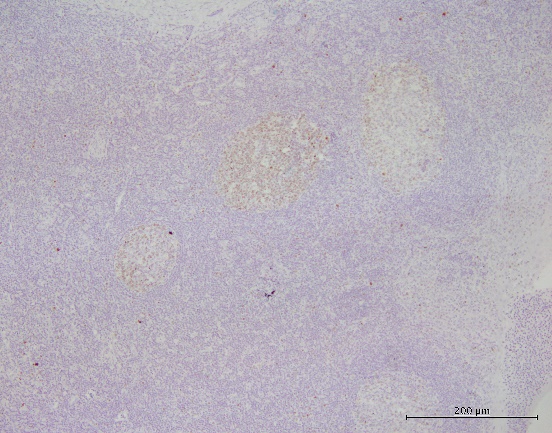

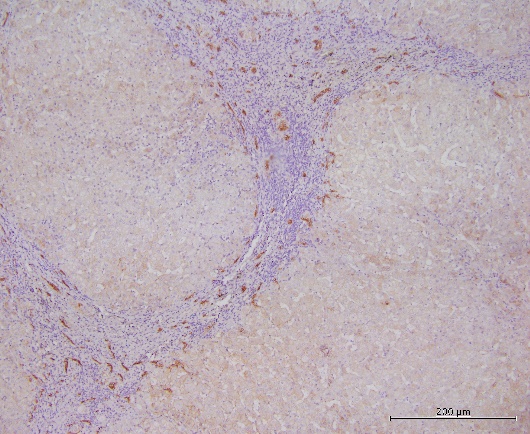

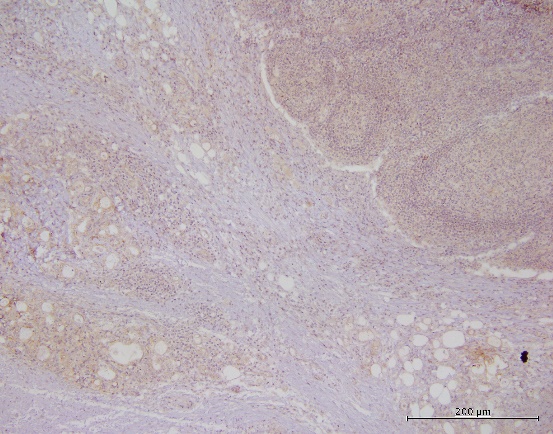


**Figure S1.** Positive control tissues for **(A)** CYP11B2, **(B)** CYP17A1, **(C)** KCNJ5, **(D)** Ki67, **(E)** β-catenin and **(F)** LHCGR. Specific and selective staining of **(A)** zona glomerulosa cells in the adrenal for CY11B2 staining, **(B)** leydig cells in the tesitis **(C)** zona glomerulosa cells in the adrenal and **(D)** germinal centers in the tonsils. Membranous expression with nuclear expression **(E)** and membranous expression **(F)** in breast carcinomas are shown.


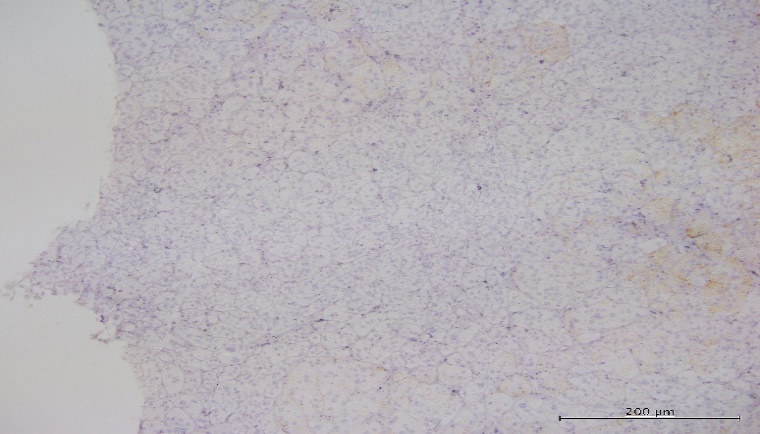

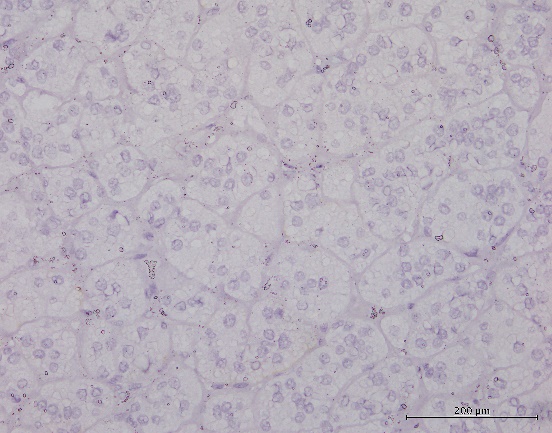


Score 0


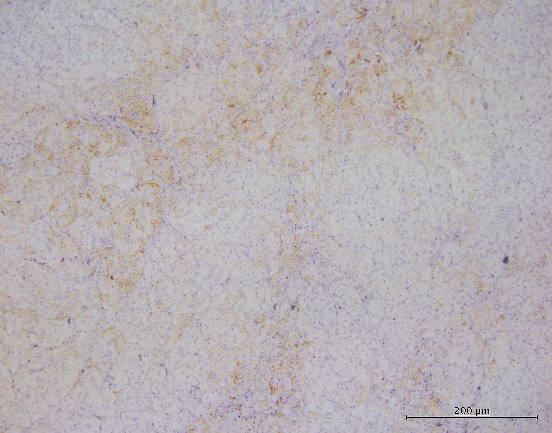

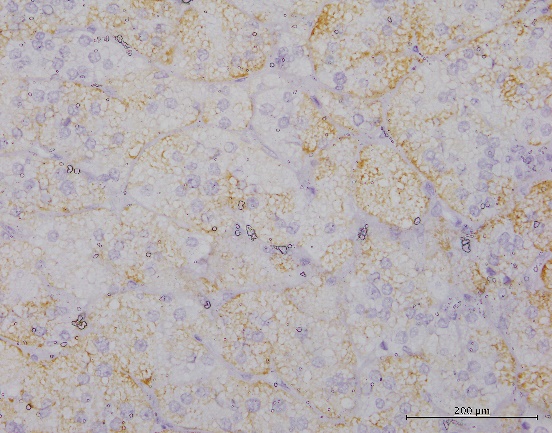


Score 3


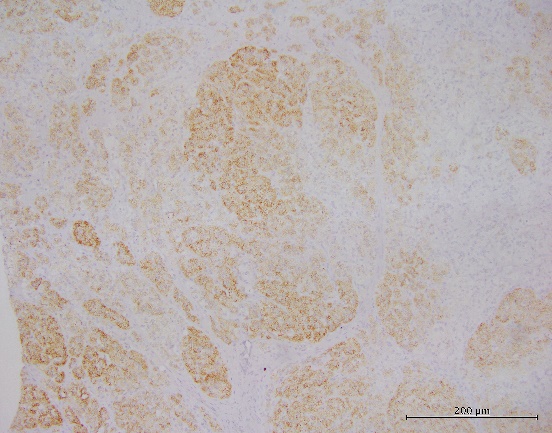

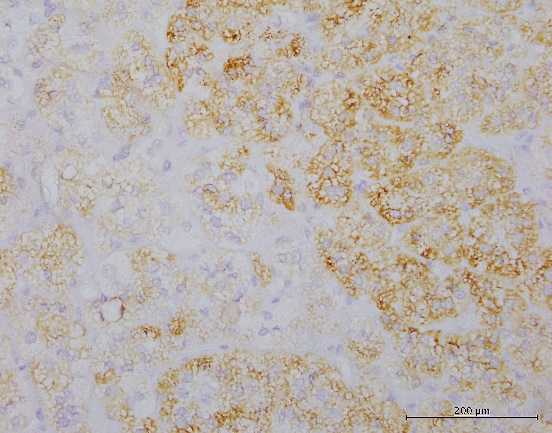


Score 6


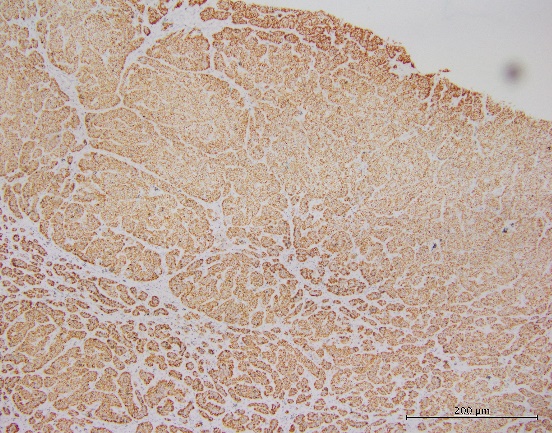

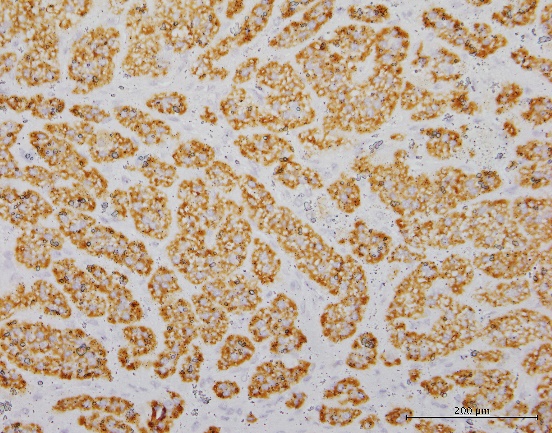


Score 10

**Figure S2**. Illustration of different immunointensities (minimum, medium and maximum) of CYP11B2 immunostaining. Magnification of 10× (left column) and 40× (right column).


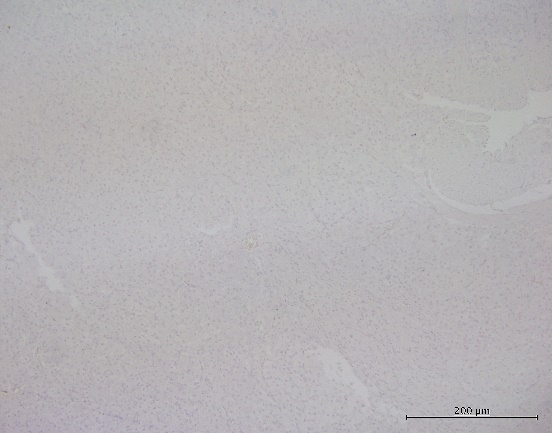

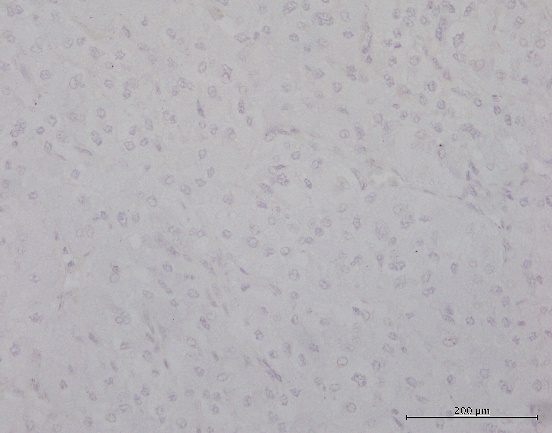

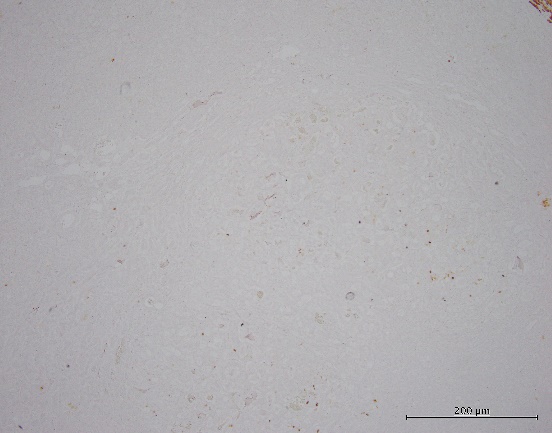

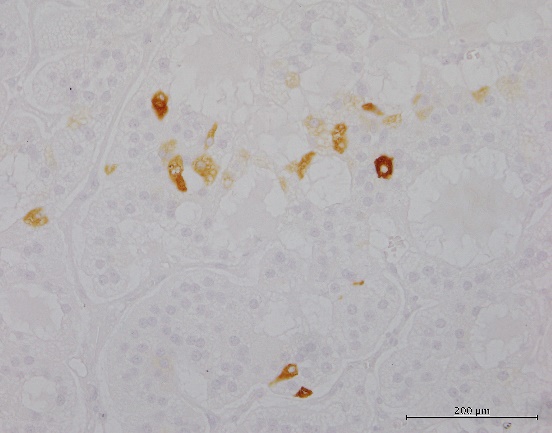

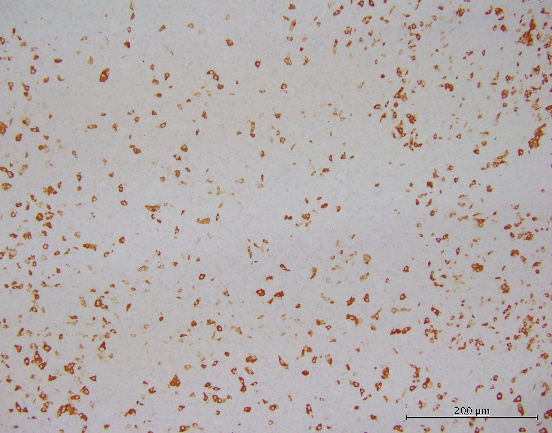

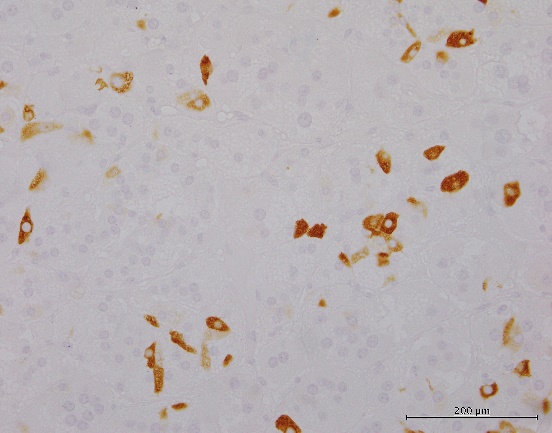

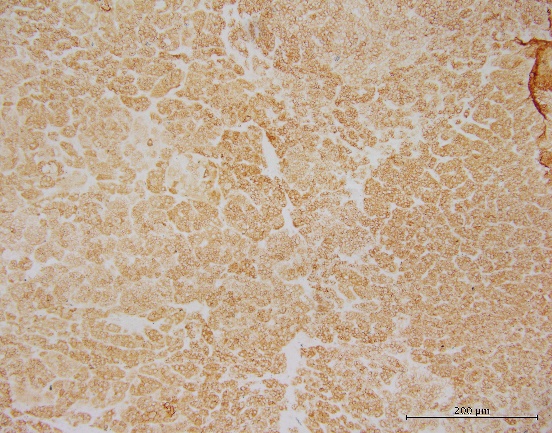

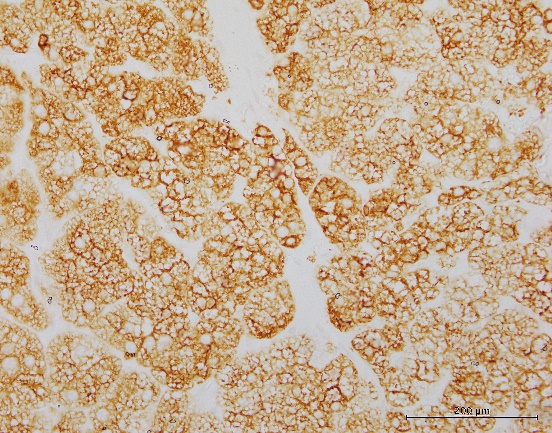


Score 0

Score 8

Score 3

Score 1

**Figure S3.** Illustration of different immunointensities (minimum, medium and maximum) of CYP17A1 immunostaining. Magnification of 10× (left column) and 40× (right column).


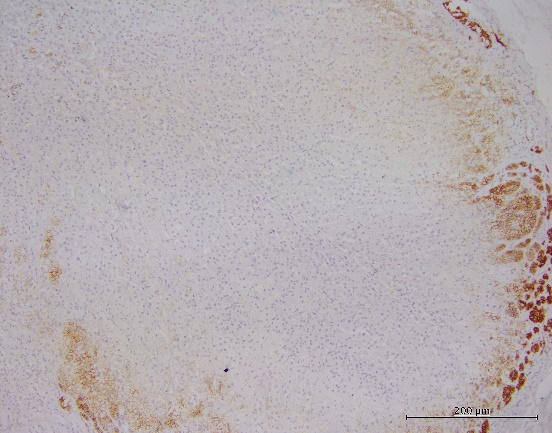

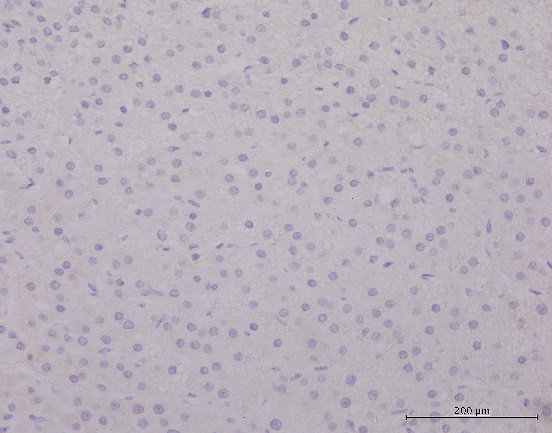


Score 0


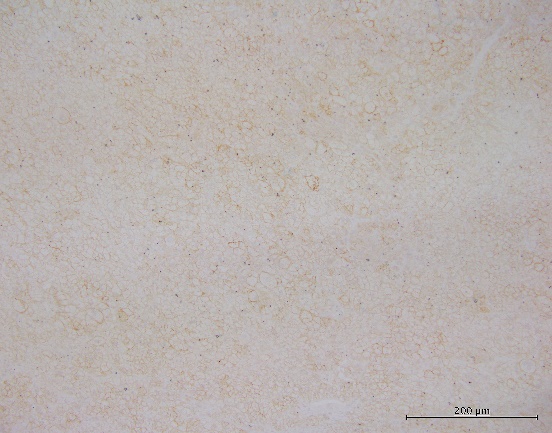

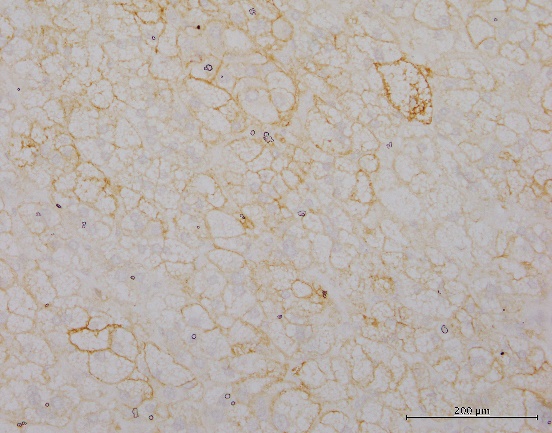


Score 3


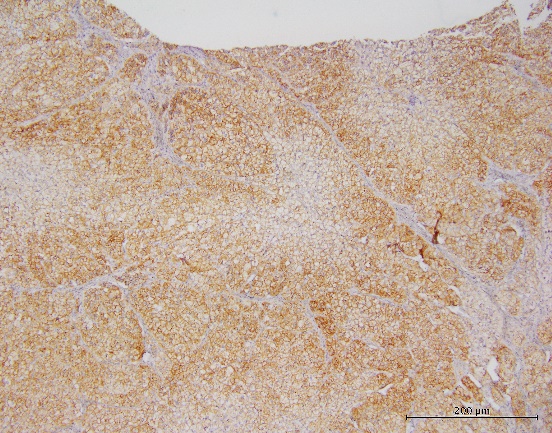

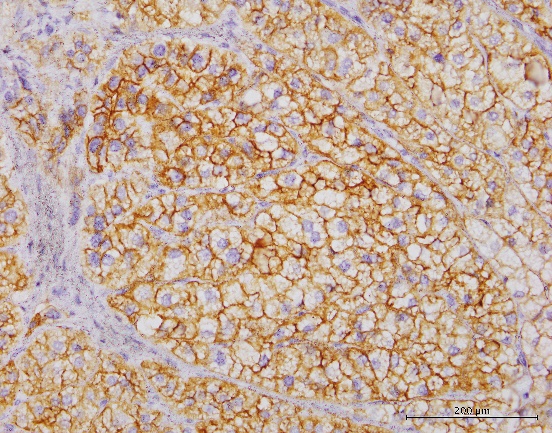


Score 7


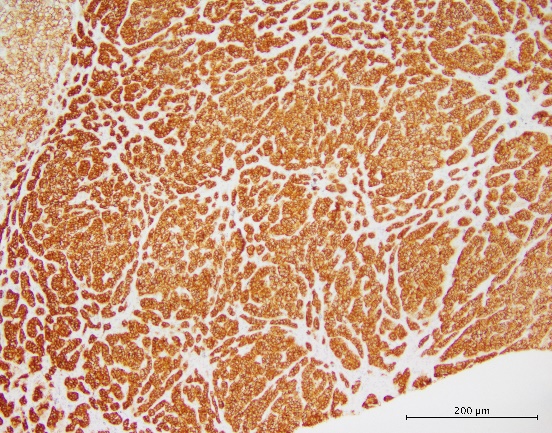

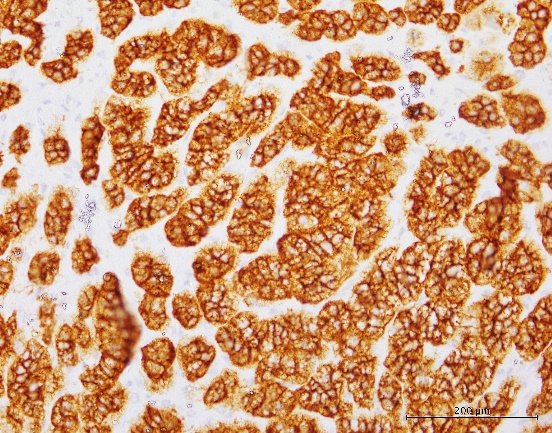


Score 10

**Figure S4.** Illustration of different immunointensities (minimum, medium and maximum) of KCNJ5 immunostaining. Magnification of 10× (left column) and 40× (right column).


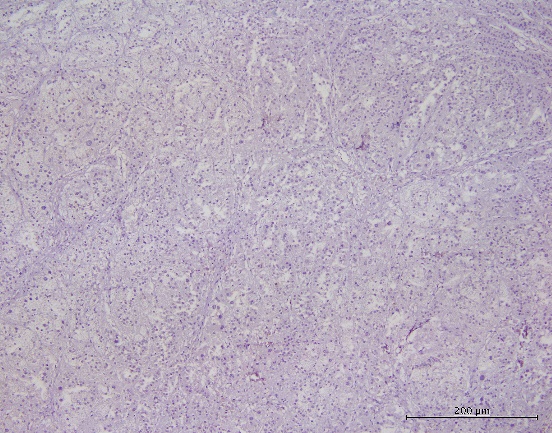

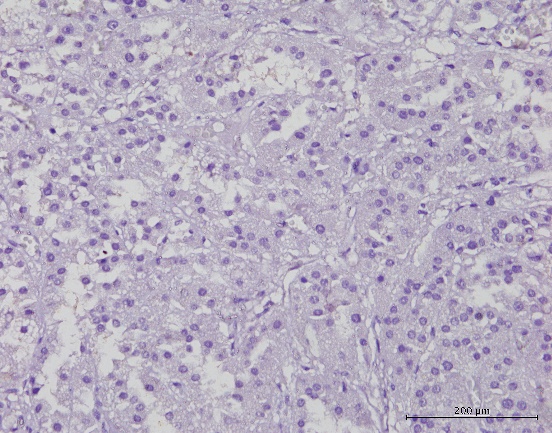


Score 0


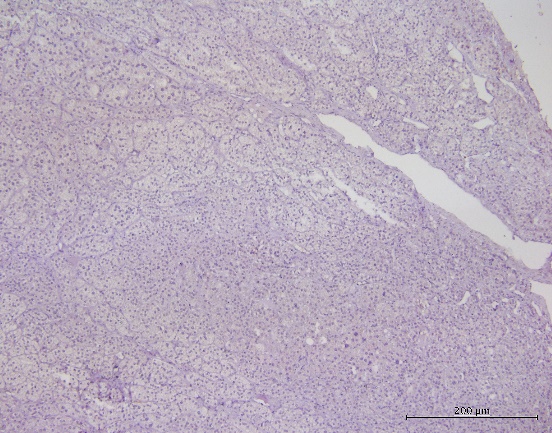

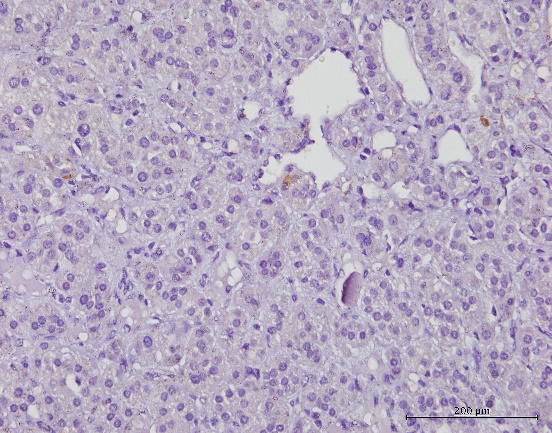


Score 1


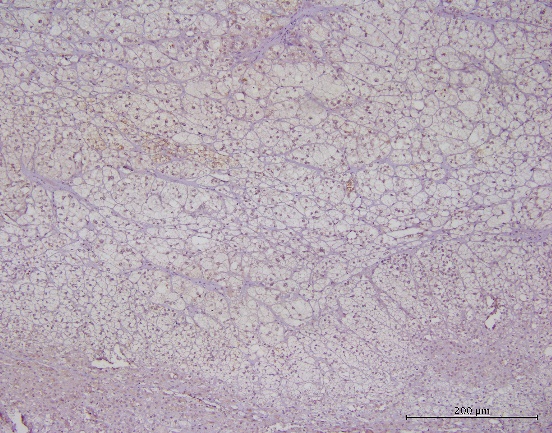

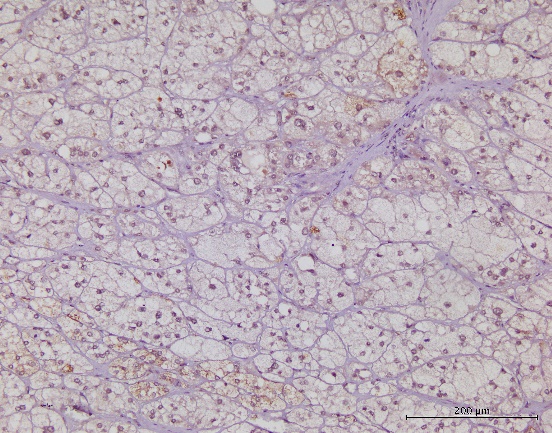


Score 3


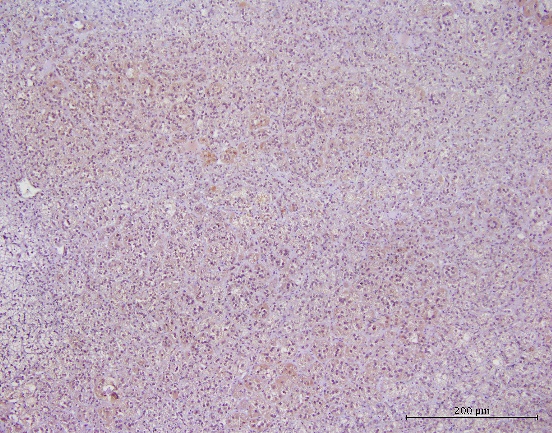

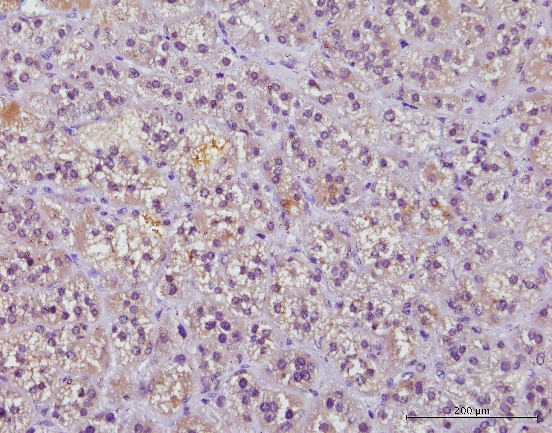


Score 4

**Figure S5**. Illustration of different immunointensities (minimum, medium and maximum) of LHCGR immunostaining. Magnification of 10× (left column) and 40× (right column).


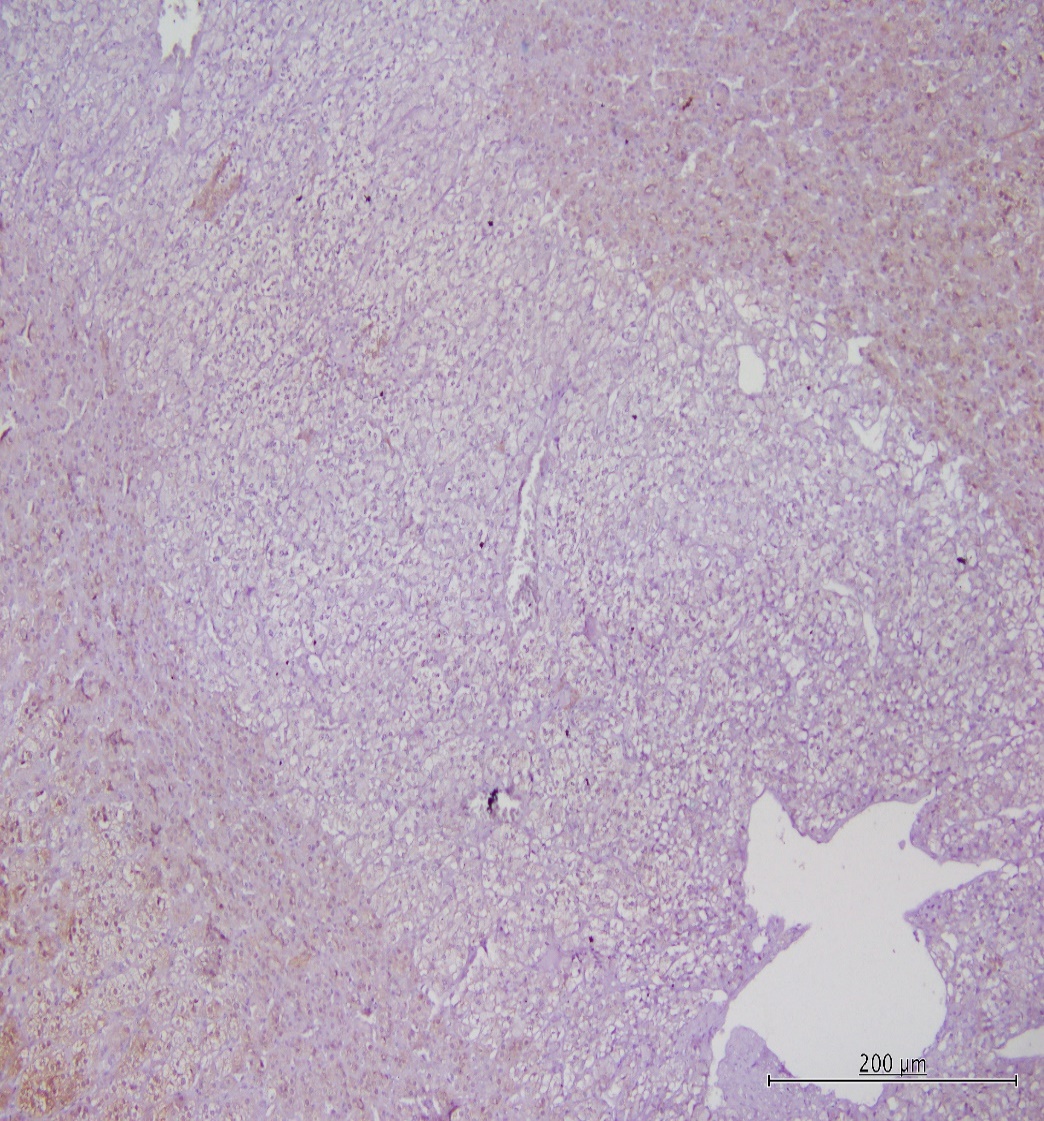

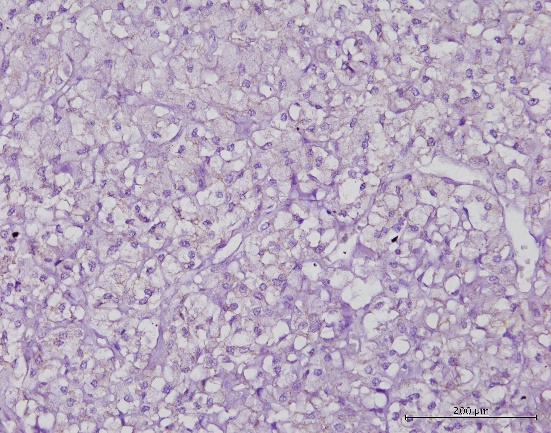


Score 0


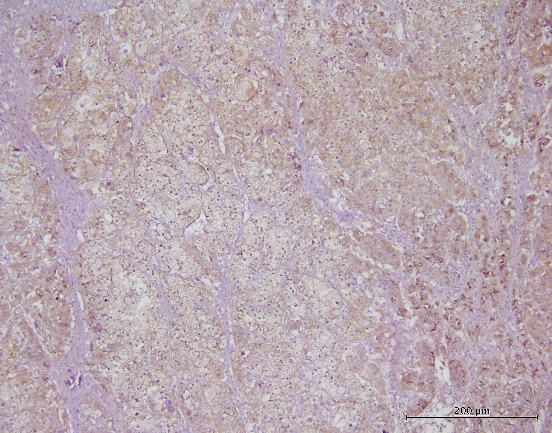

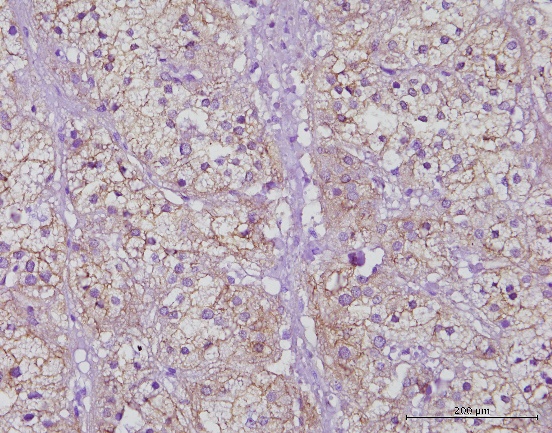


Score 1


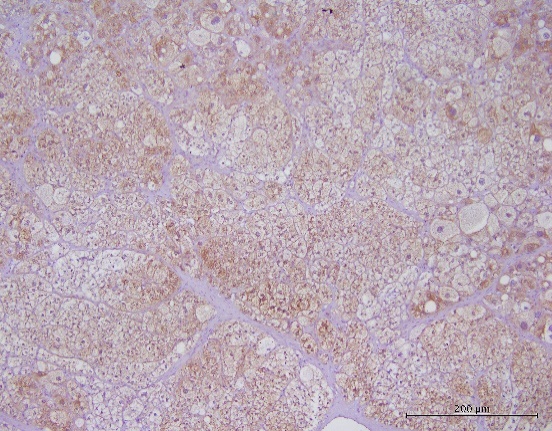

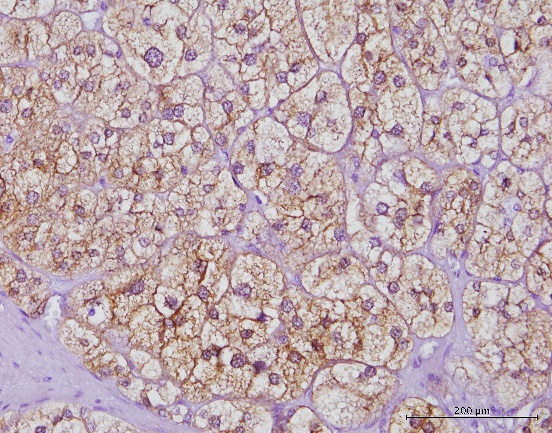


Score 3


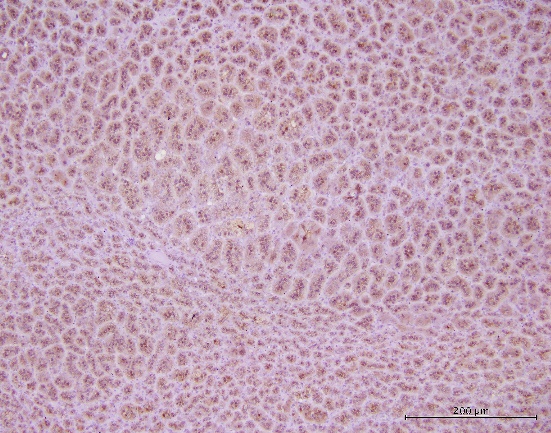

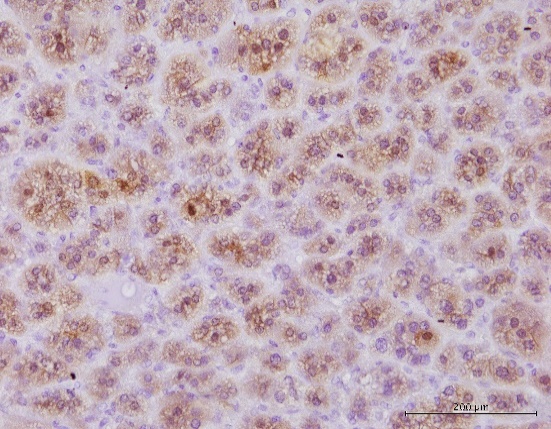


Score 4

**Figure S6**. Illustration of different immunointensities of β-catenin immunostaining. Magnification of 10× (left column) and 40× (right column).

**A**


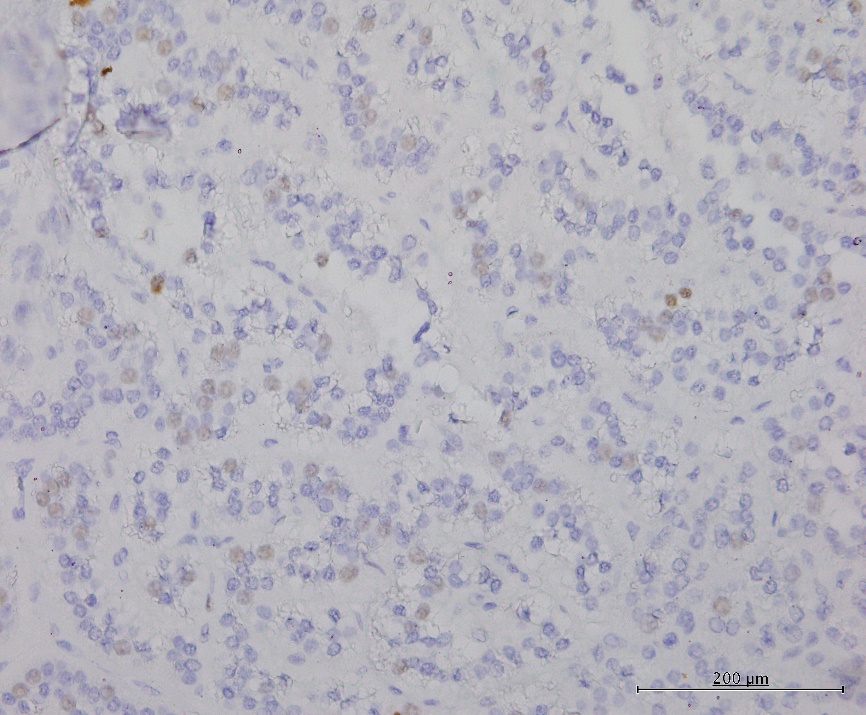


**B**


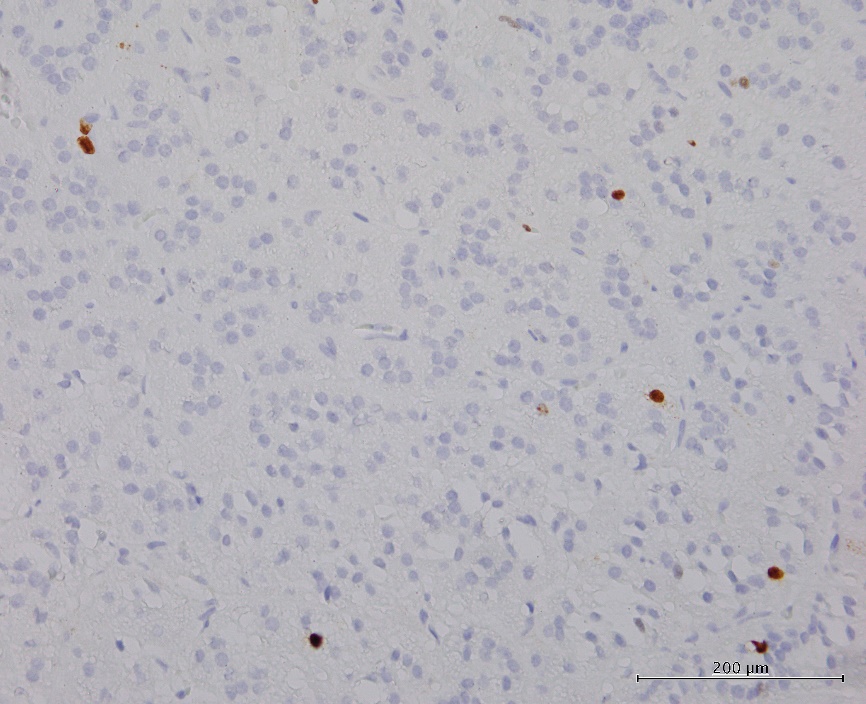


**Figure S7**. Representative moderate Ki67 staining (**A**) and intense staining of Ki67 (**B**) in APA. Yellow arrows indicated moderate staining and red arrows indicated intense staining of Ki67. Scale bars, 50μm.

**β-catenin staining**

**KCNJ5 staining**

**CYP17A1**

**staining**

**CYP11B2 staining**

***CACNA1D***

**Mutant APA**

***ATP1A1***

**Mutant APA**

***KCNJ5***

**Mutant APA**


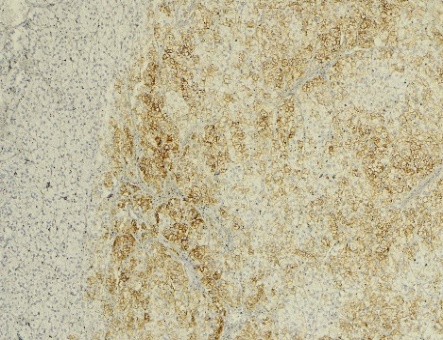


**APA**

**NA**


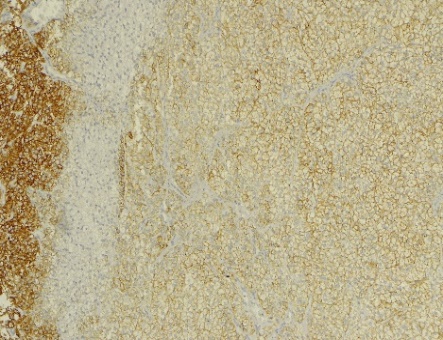


**APA**

**NA**


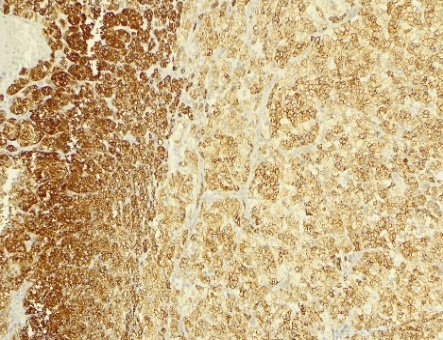


**NA**

**APA**


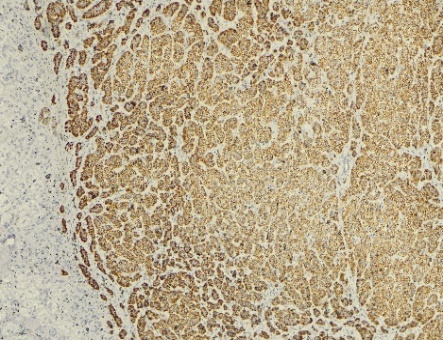


**APA**

**NA**


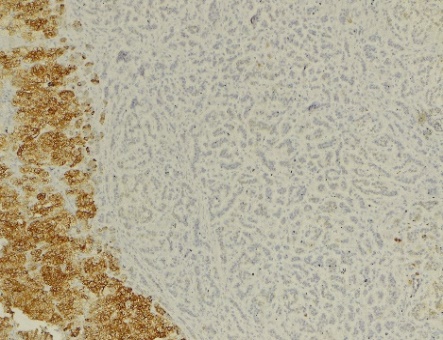


**APA**

**NA**


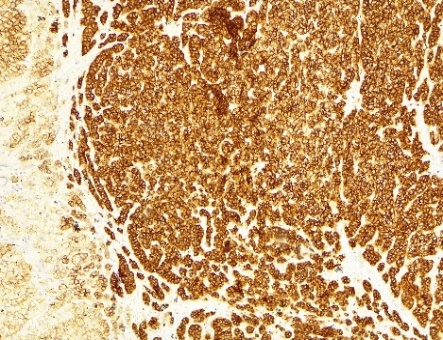


**APA**

**NA**


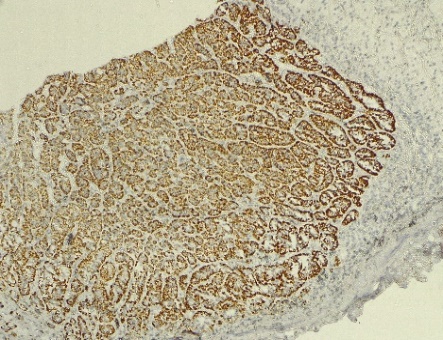


**APA**

**NA**


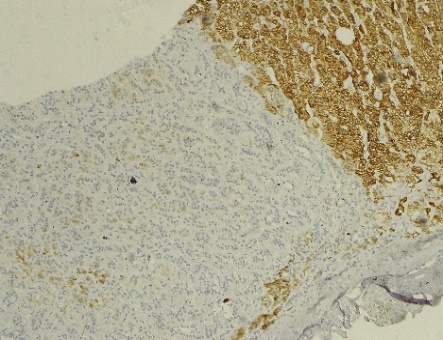


**APA**

**NA**


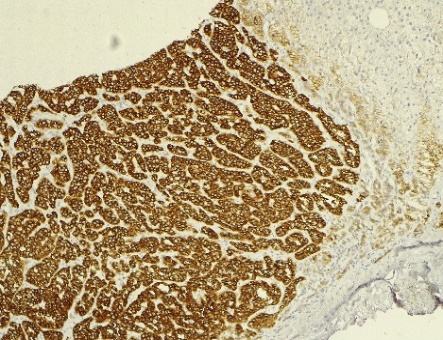


**APA**

**NA**


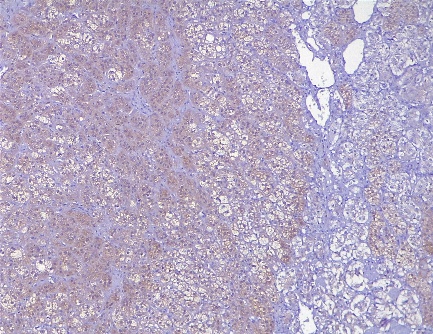


**APA**

**NA**


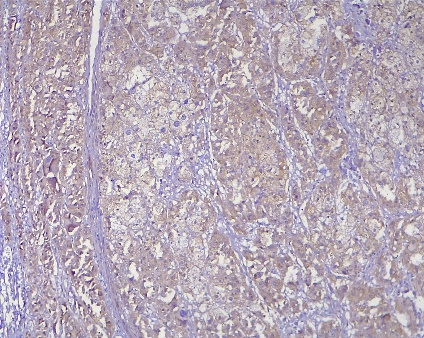


**NA**

**APA**


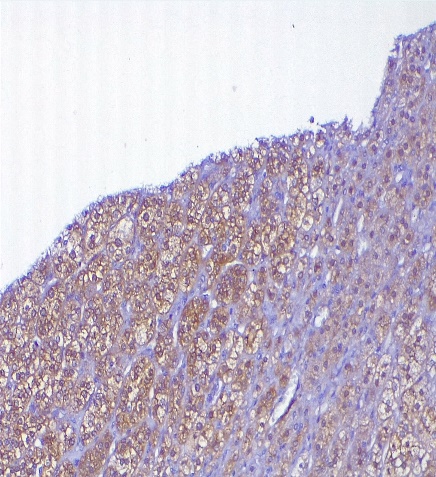


**APA**

**NA**

**Figure S8.** Representative immunohistochemical staining (CYP11B2, CYP17A1, KCNJ5 & β-catenin) in *KCNJ5*, *ATP1A1* and *CACNA1D* mutant APAs

**Ki67 staining**

**(zoom of APA)**

**LHCGR staining**

**(zoom of APA)**

**β-catenin staining**

**(zoom of APA)**

***KCNJ5***

**Mutant APA**

***ATP1A1***

**Mutant APA**

***CACNA1D***

**Mutant APA**


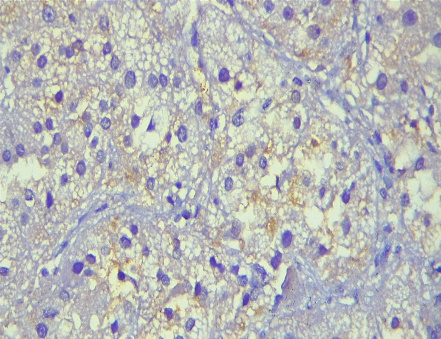

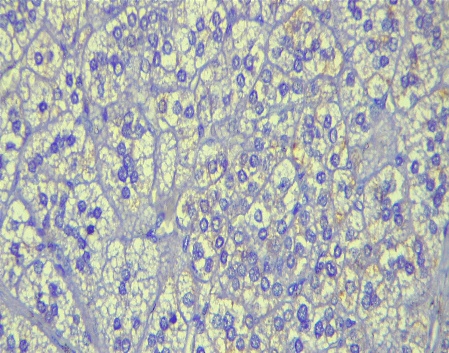

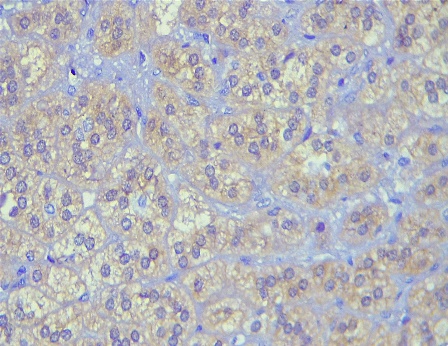

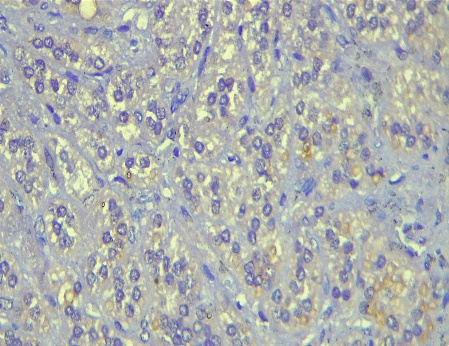

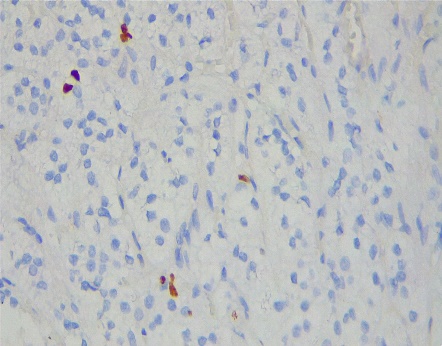

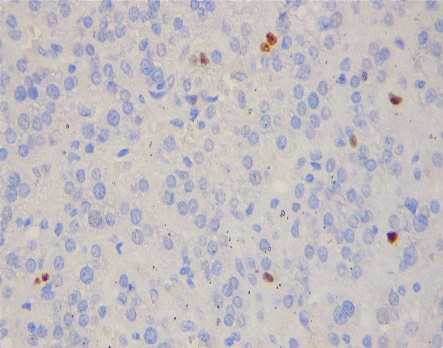

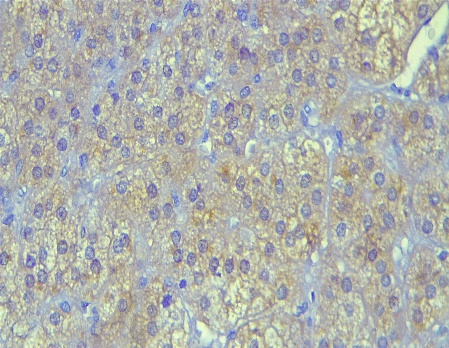

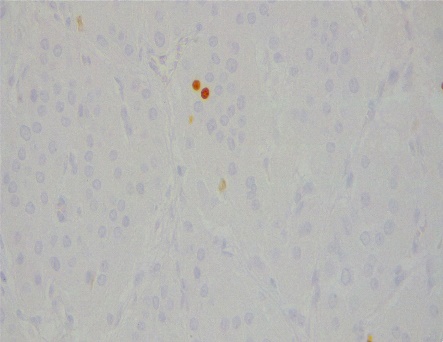

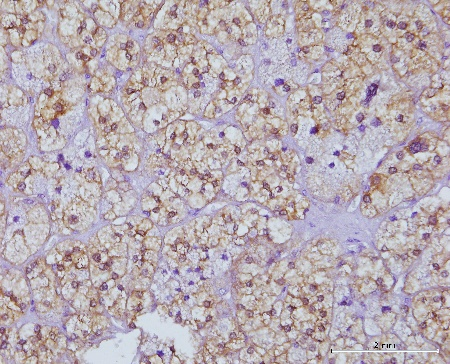


**Figure S9.** Zoom image of β-catenin, LHCGR and Ki67 in IHC staining of *KCNJ5*, *ATP1A1* and *CACNA1D* mutant APAs

***CTNNB1+CACNA1D***

**Double Mutant APA**

***CTNNB1***

**Single Mutant APA**

**β-catenin**

**IHC staining**

**KCNJ5**

**IHC staining**

**CYP17A1**

**IHC staining**

**CYP11B2**

**IHC staining**


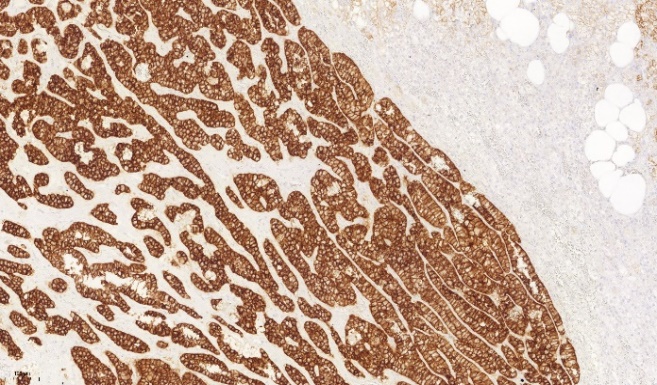


**APA**

**NA**


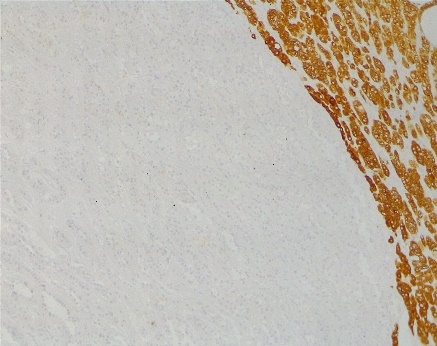


**APA**

**NA**


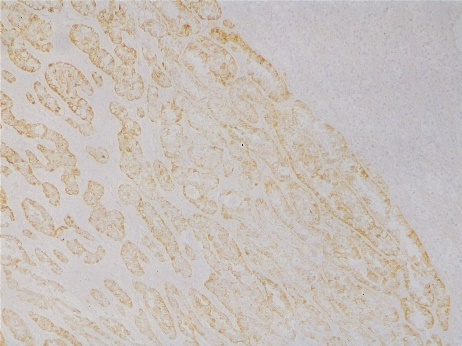


**NA**

**APA**


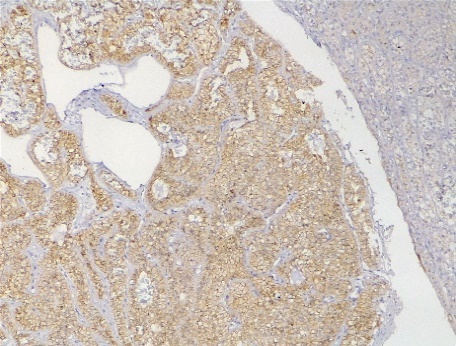


**NA**

**APA**


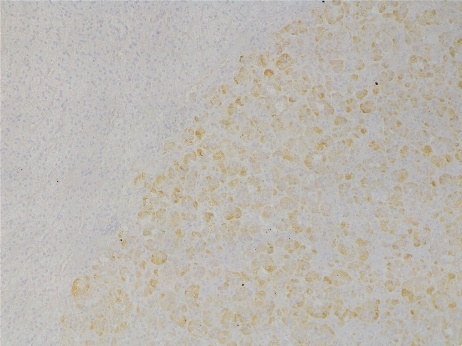


**NA**

**APA**


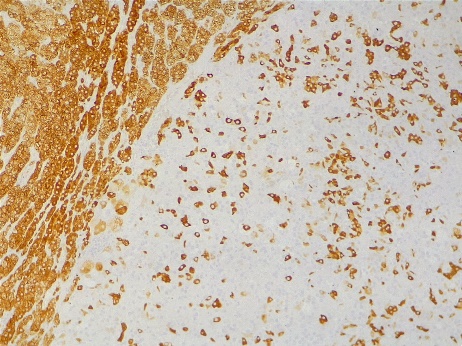


**APA**

**NA**


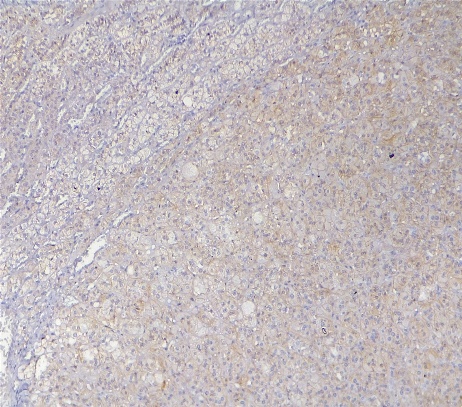


**APA**

**NA**


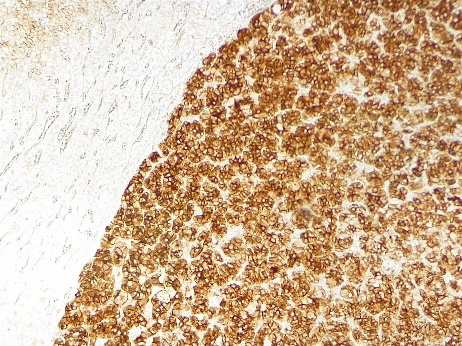


**APA**

**NA**

**Figure S10.** Comparison of IHC staining of *CTNNB1* and *CACNA1D* double mutant APA and *CTNNB1* single mutant APA (CYP11B2, CYP17A1, KCNJ5 and β-catenin IHC staining)

**CYP11B2 staining**

***ATP1A1* Mutant APA**


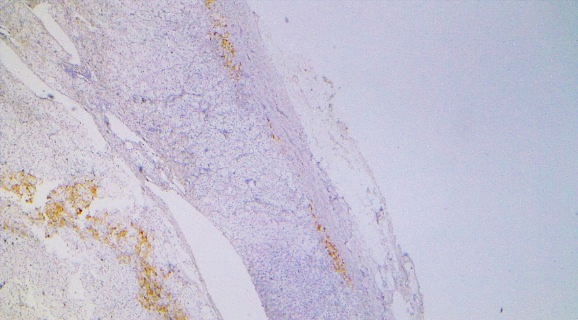

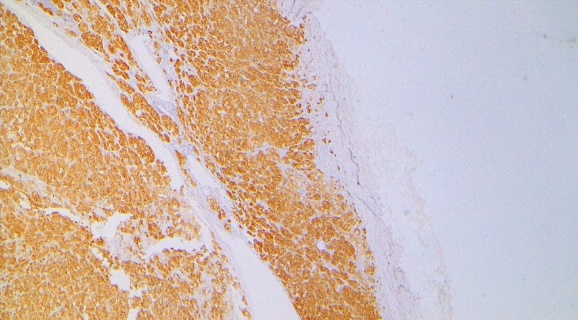

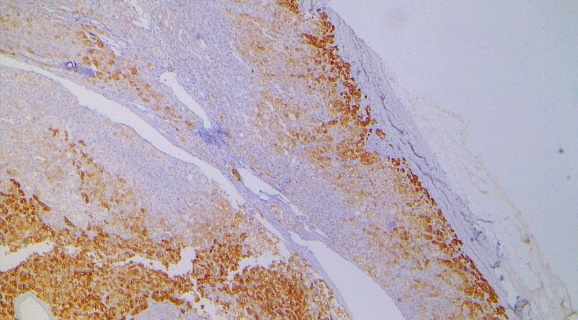


***KCNJ5* Mutant APA**

**CYP17A1 staining**

**KCNJ5 staining**


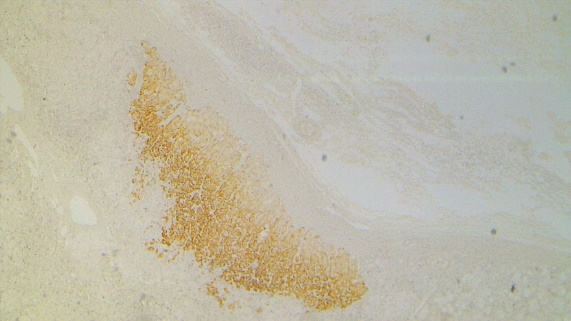

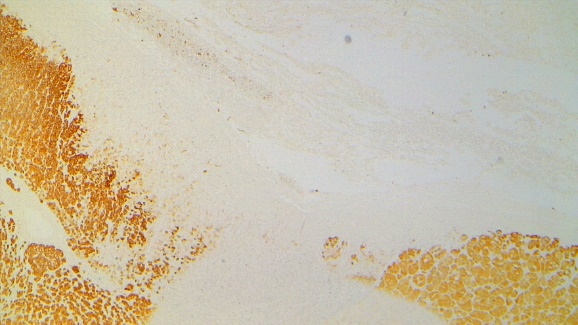

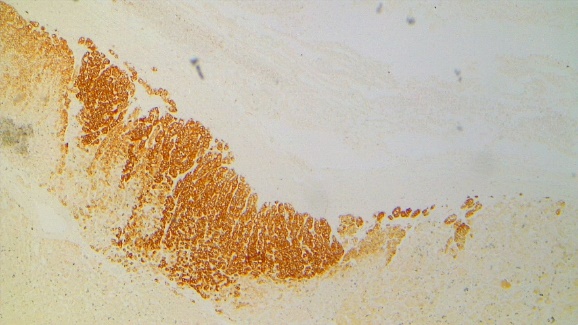


**Figure S11.** Representative images of adrenals harbouring *ATP1A1* and *KCNJ5* mutant APAs with CYP11B2 positive ZG identified by lack of CYP17A1 IHC staining and intense KCNJ5 IHC stainin
